# Linking macroecology and community ecology: refining predictions of species distributions using biotic interaction networks

**DOI:** 10.1111/ele.12770

**Published:** 2017-04-21

**Authors:** Phillip P.A. Staniczenko, Prabu Sivasubramaniam, K. Blake Suttle, Richard G. Pearson

**Affiliations:** ^1^ National Socio‐Environmental Synthesis Center (SESYNC)AnnapolisMDUSA; ^2^ Department of BiologyUniversity of MarylandCollege ParkMarylandMDUSA; ^3^ Centre for Biodiversity and Environment ResearchUniversity College LondonLondonUK; ^4^ School of Biological SciencesInstitute of Quantitative Biology, Biochemistry and BiotechnologyUniversity of EdinburghEdinburghUK; ^5^ Department of Ecology and Evolutionary BiologyUniversity of CaliforniaSanta CruzCAUSA

**Keywords:** Bayesian networks, biotic interactions, climate change, community ecology, geographical range, networks, species distribution models

## Abstract

Macroecological models for predicting species distributions usually only include abiotic environmental conditions as explanatory variables, despite knowledge from community ecology that all species are linked to other species through biotic interactions. This disconnect is largely due to the different spatial scales considered by the two sub‐disciplines: macroecologists study patterns at large extents and coarse resolutions, while community ecologists focus on small extents and fine resolutions. A general framework for including biotic interactions in macroecological models would help bridge this divide, as it would allow for rigorous testing of the role that biotic interactions play in determining species ranges. Here, we present an approach that combines species distribution models with Bayesian networks, which enables the direct and indirect effects of biotic interactions to be modelled as propagating conditional dependencies among species’ presences. We show that including biotic interactions in distribution models for species from a California grassland community results in better range predictions across the western USA. This new approach will be important for improving estimates of species distributions and their dynamics under environmental change.

## Introduction

Ecological studies at different spatial scales tend to ask different questions and use different methods. Macroecologists are traditionally concerned with species distributions, diversity and abundance at large spatial extents and coarse spatial resolutions. They typically analyse global or continental databases using geographic information systems and spatial statistics. By contrast, community ecologists ask questions related to interactions among species and ecosystem function at the smaller extents and finer resolutions of individual research sites. Community ecology has a long history of using local and often long‐term experiments, the likes of which are not feasible at larger, macroecological scales. Findings about environmental change from these two sub‐disciplines can also seem quite different. For example, studies of the ecological impacts of climate change at macro scales have revealed common and predictable trends, such as pole‐ward shifts in species distributions and advancement of spring events (Parmesan & Yohe 2003). However, experimental studies at individual field sites often show more idiosyncratic responses to environmental change, characterised by nonlinearities and feedbacks that can be difficult to predict (Suttle *et al*. 2007; Tylianakis *et al*. 2008). There is a need to reconcile such findings at different scales, which also offers the opportunity to deepen our understanding by using local scales to inform large scales and *vice versa* (Paine 2010). It is therefore crucial to develop new methodological frameworks that are able to link data across scales and build bridges between sub‐disciplines.

One area in which studies at different scales have historically been very disparate is in the use of ecological niche theory. Macroecologists tend to be schooled in the niche concepts of Grinnell (1917), who defined a species’ niche on the basis of the abiotic environmental conditions (e.g. temperature, precipitation) required to maintain a population. Community ecologists, on the other hand, tend to use the niche concept of Elton (1929), who emphasised the functional role of a species in a biotic community. In reality, both abiotic environment and biotic community impact species, and recent work has made progress integrating Grinnellian and Eltonian niche concepts to improve knowledge of how ecological systems operate across spatial scales (Soberón 2007; Peterson *et al*. 2011; Trainor & Schmitz 2014). Of particular interest is the role that biotic interactions play in determining species geographical ranges. For instance, the Eltonian Noise Hypothesis posits that species ranges at large extents and coarse resolutions are determined principally by abiotic factors, with little impact of local biotic interactions (Soberón 2007; Soberón & Nakamura 2009). Developing ways to test this hypothesis and include biotic interactions in models of species distributions will have great practical value across a range of applications, from predicting range shifts under climate change to anticipating the spread of invasive species and zoonotic diseases (Peterson *et al*. 2011).

Species distribution models (SDMs, also called ecological niche models) are widely used to estimate species ranges, typically at macroecological scales. These models use statistical associations between known occurrences and abiotic variables to project probabilities of occurrence under changed conditions (Guisan & Thuiller 2005). Several different methods have been used for SDMs (Elith *et al*. 2006), and mechanistic approaches that incorporate physiology and life history traits show potential for improving predictions (Kearney & Porter 2009; Pearson *et al*. 2014). However, current methods overwhelmingly ignore biotic interactions (Urban 2015), despite wide acknowledgement that this is a potentially severe limitation of the models (Pearson & Dawson 2003; Schmitz *et al*. 2003; Guisan & Thuiller 2005; Araújo & Luoto 2007; Kissling *et al*. 2012; Ockendon *et al*. 2014; Thuiller *et al*. 2015).

Every species, regardless of where it occurs, is linked to other species through competitive, symbiotic, consumptive or pathogenic interactions. So in all but the simplest systems, species’ fates are woven together as networks of biotic interactions. Results of early global change experiments signalled prominent roles for interactions in moderating species’ responses (Chapin *et al*. 1995; Harte & Shaw 1995). This was tested explicitly and affirmed in both laboratory (Davis *et al*. 1998; Petchey *et al*. 1999) and field experiments (Dormann *et al*. 2004; Suttle *et al*. 2007). Empirical evidence for the importance of biotic interactions in the context of environmental changes now spans a wide diversity of habitat types (Post & Pedersen 2008; Martin & Maron 2012; Barton & Ives 2014; Paul & Johnson 2014), and pronounced interaction effects are evident in all manner of species’ responses to recent environmental changes in the natural world (Edwards & Richardson 2004; Winder & Schindler 2004; Munson *et al*. 2008; Ling *et al*. 2009).

It has been questioned whether the effects of biotic interactions extend to the macroecological scales of most SDMs, and given the disconnect in scales between empirical research and macroecological modelling, it is important to ask whether biotic interactions, which occur at scales of individuals, add predictive value at much coarser resolutions and larger extents. Several tests from both modern and historical records suggest that they do (Araújo & Luoto 2007; Heikkinen *et al*. 2007; Gotelli *et al*. 2010; Hellman *et al*. 2012; Pateman *et al*. 2012; Blois *et al*. 2013; Mason *et al*. 2014) and there has been notable progress in developing frameworks to account for interactions in macroecological models (Pellissier *et al*. 2010; Fordham *et al*. 2013; Trainor *et al*. 2014).

Most attempts at integrating biotic interactions with SDMs have focused on explaining patterns of species co‐occurrence rather than modelling interactions explicitly (Araújo *et al*. 2011; Morueta‐Holme *et al*. 2016). One study compared predictions of species occurrence from SDMs that included only abiotic variables to those that also included recorded presences of other species in the community as additional explanatory variables (Giannini *et al*. 2013). Another study combined a graph theoretic, trophic interaction model with an SDM to account for differences in resource accessibility across locations when predicting the geographical distribution of a consumer species (Trainor & Schmitz 2014). Other studies have used known or expected biotic interactions to constrain the output of SDMs (Fernandes *et al*. 2013; Pellissier *et al*. 2013). A complementary line of work has used the output of SDMs to infer the structure of food webs – trophic interactions – at different locations and under different climate change scenarios (Albouy *et al*. 2014; Morales‐Castilla *et al*. 2015). In these approaches, a single species is examined at a time and only it and its interaction partners are considered in SDMs, ignoring all other biotic interactions in a community. But the outcomes of interspecific interactions produce ‘knock‐on’ effects along additional linkages, making it difficult to fully articulate or bound biotic interaction networks and therefore the task of modelling biotic interaction networks even more challenging.

Here we show how Bayesian networks (BNs, also called belief networks, Bayesian belief networks or Bayes nets) can be used to model biotic interactions in a manner that allows their direct and indirect effects to propagate among species in a community – following dynamics in nature – and, when combined with SDMs, can result in better estimates of species ranges. BNs have been applied to a wide range of problems across multiple disciplines (Jensen 2001; Neapolitan 2003; Pearl 2009) and are increasingly being used in environmental modelling and management (Uusitalo 2007; Mantyka‐Pringle *et al*. 2016) and to model secondary extinctions in food webs (Eklöf *et al*. 2013). In our case, BNs offer a way to model large numbers of interacting species simultaneously, with the flexibility to define interactions from macro‐scale presence‐absence patterning among species, from community‐level understanding of positive and negative interactions, or from a combination of the two approaches.

First, we describe our approach to modelling biotic interactions as BNs and show how to integrate this step in an existing SDM workflow. We then demonstrate our approach by applying it to a species pool of grassland plants in the western USA for which community‐level interactions are well defined from 18 years of research in a study system where all species co‐occur (Suttle *et al*. 2007; Sullivan *et al*. 2016). This record of community‐level understanding provides an opportunity to test interactions derived from statistical associations of species occurrences at macro scales for community‐level ecological realism. We find that many of the derived interactions are ecologically realistic, and that including biotic interactions in SDMs with this approach improves predictions of species occurrence for this example system. Finally, we use future climate scenarios to show how species’ predicted distributions in 2050 are refined when biotic interactions are included in models.

## Incorporating Biotic Interactions in Species Distribution Models

### Overview

BNs are probabilistic graphical models that represent a set of random variables as nodes and conditional dependencies between random variables as directed edges between nodes (Pearl 1988; Koller & Friedman 2009). Random variables in our BNs are probabilities of species occurrence and biotic interactions are modelled as positive and negative conditional dependencies among random variables. In this way, the probability of species occurrence from an SDM at a particular location can be modified up or down given the expected presence of any interaction partners and their combined effect on the focal species. When computing modified occurrence probabilities using BNs, the effects of conditional dependencies are propagated through the entire interaction network, meaning that new predictions reflect all biotic interactions in a community.

Conditional dependencies can represent a variety of biological processes and relationships among species. For example, competition can be described by negative conditional dependencies that decrease occurrence probabilities in line with how many competitors are present at a given location. Nitrogen fixation among plants can be described by positive conditional dependencies that increase occurrence probabilities if nitrogen‐fixing species are also present. Conditional dependencies can also reflect the effects of shared and mutually exclusive habitat suitability among species. For example, positive conditional dependencies can capture environmental conditions that are suitable for multiple species – a feature that is not possible with conventional SDMs designed solely for individual species. Although shared habitat suitability can hardly be considered a *biotic* interaction, there remains value in using conditional dependencies in this way to leverage otherwise ignored information about one species to improve predictions for other species in the community.

When incorporating biotic interactions in SDMs using BNs, we start with the output of a conventional SDM, which we refer to as *priors*. Priors are then combined with a BN to give *posteriors*. In the description that follows, we write the prior of species *i* at location *x* as $\pi_{i,x}$ and the posterior as $p_{i,x}$. The linking of SDMs and BNs is clear and elegant when the output of an SDM is the probability of occurrence of a species. However, SDM outputs are rarely true probabilities of occurrence; rather, outputs are typically habitat suitability values that are normalised between zero and one (Gotelli & Stanton‐Geddes 2015). We can still use the mechanics of BNs in such cases, but must be careful to think in terms of conditional dependencies as modifying habitat suitability values up or down depending on the combined effect of multiple biotic interactions.

### Modelling biotic interactions using Bayesian networks

In a BN, the nodes (random variables) and edges (conditional dependencies) form a graphical structure that is a directed acyclic graph (DAG), which is defined by two conditions: (1) edges are directed; and (2) the graph is acyclic (i.e. it is not possible, starting from a given node, to travel along a set of directed edges and return to the starting node; Thulasiraman & Swamy 1992). Although it is common to see directed edges interpreted as causal relationships, this is not always justified (Thulasiraman & Swamy 1992; Spirtes *et al*. 2000; Dawid 2008).

**Figure 1:**
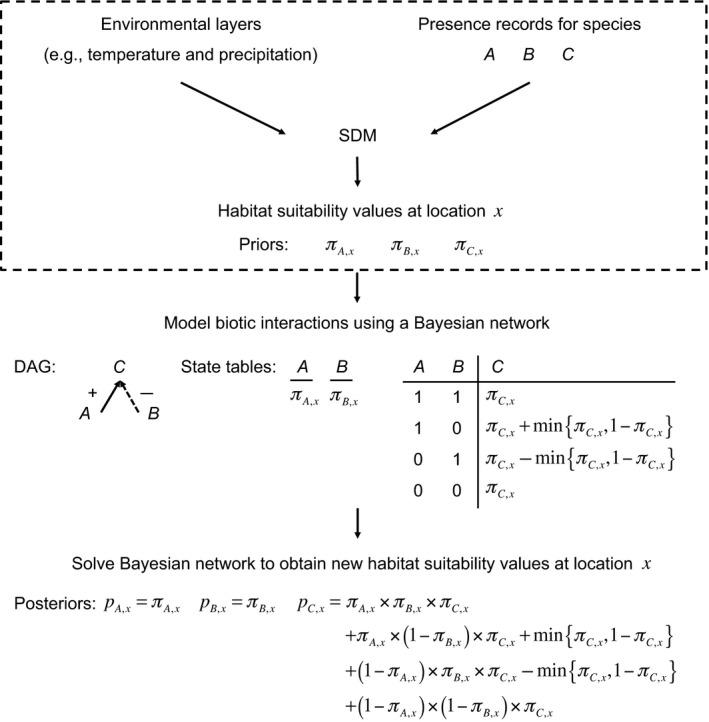
Workflow for including biotic interactions in species distribution models (SDMs): an example with three species. In a conventional SDM workflow (dashed box), data on environmental conditions and species presences are combined to predict habitat suitability across a geographical extent for species *A*,* B* and *C*, separately. When modelling biotic interactions using a Bayesian network (BN), we refer to the output of conventional SDMs as *priors*. The BN network comprises a directed acyclic graph (DAG) that describes the pattern of positive and negative conditional dependencies among species in a community, and a corresponding set of state tables that describe how each species is affected by the presence of other species (here, the presence of *C* is increased by the presence of *A* but decreased by the presence of *B*). The process of solving the BN transforms priors into *posteriors*, which now incorporate the effect of biotic interactions on habitat suitability. Note that the state table entries in this example are consistent with all three AND, OR and SUM models.

In our approach, nodes are species and directed edges are biotic interactions: nodes represent the probability of species occurrence at a particular location and directed edges represent the effect on occurrence of one species on another (Fig. 1). Note that we are dealing with Boolean random variables because the outcome at a particular location is either present (1) or absent (0). By way of illustration, consider three species: *A*,* B* and *C*. The presence of *C* is positively affected by *A* but negatively affected by *B*, which can be represented by *A* → *C* ↚ *B*; where *C* is referred to as the *child* node of *parent* nodes *A* and *B*, which, as *A* and *B* have no conditional dependencies themselves, would also be called *root* nodes. This DAG indicates that the probability of occurrence of *C* at a particular location is conditionally dependent on whether species *A* and *B* are present at the location. There are $2^{2}\;=\;4$ possibilities: *A* and *B* are both absent; *A* is present but *B* is absent; *A* is absent but *B* is present; and *A* and *B* are both present. The conditional probabilities associated with these four possibilities together comprise the state table for species *C*. The combination of a DAG and associated state tables (also known as conditional probability tables) for each species is a BN. A worked example of solving this BN is in Box 1.

Box 1 **A worked example of solving a Bayesian network with three species**

Consider the following Bayesian network: *A* → *C* ↚ *B*; where the presence of species *C* is positively affected by *A* but negatively affected by *B*.

At location *x* = 1, assume that the prior probability of occurrence of *C* is $\pi_{C,1}\;=\;0.4$ and its conditional probabilities are 0.4 when *A* and *B* are both absent; 0.8 when *A* is present but *B* is absent; 0 when *A* is absent but *B* is present; and 0.4 when *A* and *B* are both present, i.e. the positive influence of *A* balances the negative influence of *B*. As species *A* has no conditional dependencies, we can assume it has a single probability of occurrence at the location, say, $\pi_{A,1}\;=\;0.7$; similarly, we can assume *B* also has a single probability of occurrence, say, $\pi_{B,1}\;=\;0.2$. The probability of occurrence of *C* including the effect of biotic interactions is then $p_{C,1}\;=\;\left(1\;-\;0.7\right)\;\times \;\left(1\;-\;0.2\right)\;\times \;0.4\;+\;0.7\;\times \;\left(1\;-\;0.2\right)\;\times \;0.8\;+\;\left(1\;-\;0.7\right)\;\times \;0.2\;\times \;0.0+\;0.7\;\times \;0.2\;\times \;0.4\;=\;0.096\;+\;0.448\;+0\;+\;0.056\;=\;0.6$. In this example, the prior probabilities of occurrence $\pi_{A,1}\;=\;0.7$, $\pi_{B,1}\;=\;0.2$ and $\pi_{C,1}\;=\;0.4$ combine with the BN to give new, posterior probabilities of occurrence $p_{A,1}\;=\;0.7$, $p_{B,1}\;=\;0.2$ and $p_{C,1}\;=\;0.6$.

If the effects of *A* and *B* are switched, i.e. *A* ↛ *C* ← *B*, then the posterior for *C* becomes $p_{C,1}\;=\;0.096\;+\;0.7\times \left(1\;-\;0.2\right)\;\times \;0\;+\;\left(1\;-\;0.7\right)\;\times \;0.2\;\times \;0.8\;+\;0.056\;=\;0.096\;+\;0\;+\;0.048\;+\;0.056\;=\;0.2$, which is lower than before because the negative effect on *C* is now associated with *A*, which is more likely to be present than *B*.

As an example of propagating conditional dependencies, now assume that the presence of *A* positively affects the presence of *B*, which, in turn, positively affects the presence of *C*:* A* → *B* → *C*. Assume that the presence of *A* doubles the probability of occurrence of *B*, and similarly for the effect of *B* on *C*. As above, let the priors at the given location be $\pi_{A,1}\;=\;0.7$, $\pi_{B,1}\;=\;0.2$ and $\pi_{C,1}\;=\;0.4$. With this new BN, the posteriors are $p_{A,1}\;=\;0.7$, $p_{B,1}\;=\;0.7\;\times \;\left(2\;\times \;0.2\right)\;+\;\left(1\;-\;0.7\right)\;\times \;0.2\;=\;0.34$ and $p_{C,1}\;=\;0.34\;\times \;\left(2\;\times \;0.4\right)\;+\;\left(1\;-\;0.34\right)\;\times \;0.4\;=\;0.536$. Although the principle remains the same for communities with a greater number of species and more complex pattern of interactions, solving BNs by hand quickly becomes unwieldy. Fortunately, BNs can be solved numerically very efficiently (Thulasiraman & Swamy 1992). Example R code for solving BNs is in Appendix S1.

In practice, specifying a BN involves five main considerations. (1) Inclusion: Should a biotic interaction be modelled in the BN? (2) Direction: What is the net effect of the biotic interaction on the two interacting species, i.e. should *A* → *B* or *A* ← *B* be used? (3) Sign: Does the biotic interaction have a positive or negative effect on the presence of the affected species? (4) Strength: How much does the biotic interaction modify the presence of the affected species? (5) Combination: How do multiple biotic interactions combine to modify the presence of the affected species? These five considerations sort into two main tasks: specifying the graphical structure of the BN (considerations 1, 2 and 3) and specifying rules for filling the state table entries defined by the graphical structure (considerations 4 and 5).

### Specifying the graphical structure of a Bayesian network

The graphical structure of a BN is the particular arrangement of nodes and edges used to represent biotic interactions. The arrangement must be a DAG for the BN to be solvable; and although feedback cycles are common in ecology, it is often straightforward to organise conditional dependencies in such a way as to avoid cycles in BNs without sacrificing too much biological realism. There are two general approaches for determining which biotic interactions to include in a BN: a computer‐aided optimisation process that considers all possible species pairings based on macroecological occurrence data, or direct selection of interactions using data from community ecology.

The optimisation approach involves trialling multiple combinations of biotic interactions in order to find the combination that maximises an objective function. The area under the receiver operator curve (AUC) statistic is a familiar option for an objective function as it is widely used to measure the ability of an SDM to discriminate between known presences and absences (with presence‐only data, absences are random locations drawn from the geographical extent under investigation; Phillips *et al*. 2006). Once an objective function has been chosen, a variety of algorithms can be used for optimisation, ranging from simple Hill‐Climbing searches (Skiena 2008) to more involved genetic algorithms (Holland 1975). Note that exhaustive searches are impractical as the number of distinct DAG topologies can be large even when there are few nodes (Robinson 1973).

Alternatively, interactions can be specified based on community‐level understanding, although suitable data sets are rare given the effort, time and cost required to collect comprehensive community data. If such data are available, however, then the first step is to identify a set of candidate biotic interactions for inclusion in the BN. A subset can then be selected based on each candidate interaction's perceived importance for the study region of interest. Among this subset, a direction and sign must be associated with each interaction (strictly, sign is not necessary for specifying the graphical structure, but as the effect of a biotic interaction is closely linked with its type then it makes sense for the decision about sign to be made at this stage). Note that decisions about sign and direction are not always straightforward. With a trophic interaction in which *B* eats *A*, for example, on the one hand the presence of *B* has a negative effect on *A* but on the other hand the presence of *A* has a positive effect on *B*; in such cases, to avoid directed cycles (i.e. *A* ⇌ *B*) it is necessary to pick the direction and sign combination that is expected to have the greatest impact on species occurrence. After a DAG has been specified, the size of state tables is fixed and the next task is to fill table entries with values.

### Specifying rules for filling state table entries

A comprehensive but time‐consuming approach to filling state table entries involves determining biologically motivated values for each state table entry individually. We refer to this approach as the FULL model. This model is unlikely to be practically feasible for all but the smallest systems because a species with *N* conditional dependencies has $2^{N}$ entries in its state table. One must therefore devise rules for simplifying the process of filling state table entries. Here, we propose three models, which we refer to as AND, OR and SUM (Box 2).

Box 2 **Simple models for filling Bayesian network state table entries**

Consider a Bayesian network with four species that has a graphical structure with two positive and one negative biotic interactions: *A* → *D*,* B* → *D* and *C* ↛ *D*. The state table for *D* has $2^{3}\;=\;8$ entries that must be filled individually when using the FULL model. We can simplify the process of filling state table entries by using an AND, OR or SUM model. With the AND model (eqn 1), species *A* and *B* must both be present to increase the presence of *D*. With the OR model (eqn 2), either *A* or *B* must be present to increase the presence of *D*. With the SUM model (eqn 3), the effect of positive conditional dependencies increases with the number of species that are present, which means there are more distinct state table entries compared to AND and OR models. Schematically, we can illustrate the differences among the three models using the state table (also known as a conditional probability table) for species *D*, with no change in the expected presence of *D* represented by ‘∼’, small increases and decreases by ‘↑’ and ‘↓’, respectively, and large increases and decreases by ‘↑↑’ and ‘↓↓’:

**Table:** 

A	B	C	$D_{\mathrm{AND}}$	$D_{\mathrm{OR}}$	$D_{\mathrm{SUM}}$
1	1	1	∼	↑↑	↑
1	1	0	↑↑	↑↑	↑↑
1	0	1	∼	∼	∼
1	0	0	∼	↑↑	↑
0	1	1	∼	∼	∼
0	1	0	∼	↑↑	↑
0	0	1	↓↓	↓↓	↓
0	0	0	∼	∼	∼

The main question when deciding whether to simplify or not is ‘Does species identity matter?’ Or put in more accommodating terms: ‘Can we reasonably assume that the effects of biotic interactions are in some ways substitutable with one another?’ If species identity truly matters then the effect of each state must be determined individually, and only the FULL model is justified. But if there is some flexibility then a simpler model can be used to fill state table entries.

The two simplest models are AND and OR. Analogous to Boolean logic, all species must be present for conditional dependencies to have an effect with the AND model, but only one species must be present with the OR model. Formally, we can fill state table entries for the AND and OR models using the following rules. For species *i*, let the total number of positive conditional dependencies be $n_{+,i}^{\max}$ and the total number of negative conditional dependencies be $n_{-,i}^{\max}$. Given a particular state table entry, let the number of species present with positive conditional dependencies be $n_{+,i}$ and with negative conditional dependencies be $n_{-,i}$. With the AND model, fill the entry as

(1) $$\begin{array}{cc} \pi_{i,x} & +\text{min}\left\{\pi_{i,x},1-\pi_{i,x}\right\}\;\text{if}\;n_{+,i}=n_{+,i}^{\max}\;\text{and}\;n_{-,i}=0\\ \pi_{i,x} & -\text{min}\left\{\pi_{i,x},1-\pi_{i,x}\right\}\;\text{if}\;n_{+,i}=0\;\text{and}\;n_{-,i}=n_{-,i}^{\max}\\ & \pi_{i,x}\;\text{otherwise} \end{array}$$

With the OR model, fill the entry as

(2) $$\begin{array}{cc} \pi_{i,x}+\text{min}\left\{\pi_{i,x},1-\pi_{i,x}\right\}\; & \text{if}\;n_{+,i}-n_{-,i}>0\\ \pi_{i,x}-\text{min}\left\{\pi_{i,x},1-\pi_{i,x}\right\}\; & \text{if}\;n_{+,i}-n_{-,i}\;<\;0\\ \pi_{i,x}\; & \text{if}\;n_{+,i}-n_{-,i}=0 \end{array}$$

These two sets of rules make three important assumptions: (1) the identity of a species does not alter the effect of a biotic interaction, only whether the conditional dependency is positive or negative; (2) positive and negative conditional dependencies have the same magnitude of effect but in opposite directions, i.e. one positive conditional dependency cancels out one negative conditional dependency; and (3) the net number of positive or negative conditional dependencies is in itself unimportant, only whether the net value is at a maximal number (AND model) or whether there is a majority of positive or negative conditional dependencies (OR model). The term $\text{min}\{\pi_{i,x},\;1\;-\;\pi_{i,x}\}$ ensures that the effect of biotic interactions is bound from above and below. For example, with the OR model if $\pi_{i,x}\;=\;0.4$ then the entry for net positive conditional dependencies is 0.4 + 0.4 = 0.8 and for net negative conditional dependencies is 0.4 − 0.4 = 0, while if $\pi_{i,x}\;=\;0.75$ then likewise entries are 0.75 + 0.25 = 1 and 0.75 − 0.25= 0.5, respectively.

The next level up in complexity is the SUM model, in which the effect of conditional dependencies increases with the number of species that are present. Let us introduce for clarity two new variables: $g_{+,i}\;=\;\frac{n_{+,i}}{\text{max}\{n_{+,i}^{\max},n_{-,i}^{\max}\}}$ and $g_{-,i}\;=\;\frac{n_{-,i}}{\text{max}\{n_{+,i}^{\max},n_{-,i}^{\max}\}}$. With the four‐species system, the state (*A*,*B*,*C*) = (1,1,1) has $g_{+,D}\;=\;\frac{2}{2}\;=\;1$ and $g_{-,D}\;=\;\frac{1}{2}\;=\;0.5$, while the state (1,0,0) has $g_{+,D}\;=\;\frac{1}{2}\;=\;0.5$ and $g_{-,D}\;=\;\frac{0}{2}\;=\;0$. For a given state, we can model the combined effect of multiple biotic interactions by using the cumulative distribution function of a beta distribution to map values for $g_{+,i}$ and $g_{-,i}$ to an aggregate effect on species *i* (Eklöf *et al*. 2013). With the SUM model, fill the entry as

(3) $$\begin{array}{cc} \pi_{i,x}+ & \;\text{min}\left\{\pi_{i,x},1-\pi_{i,x}\right\}\frac{B(g_{+,i}\;;\alpha ,\beta )}{B(\alpha ,\beta )}\\ & -\text{min}\left\{\pi_{i,x},1-\pi_{i,x}\right\}\frac{B(g_{-,i}\;;\alpha ,\beta )}{B(\alpha ,\beta )} \end{array}$$

where B(α,β) is the beta function, $B(g_{+,i}\;;\alpha ,\;\beta )$ and $B(g_{-,i}\;;\alpha ,\;\beta )$ are incomplete beta functions, and α and β are shape parameters. In eqn 3, the additive effect of biotic interactions can be linear (α = β = 1), sigmoidal (α > 1, β > 1), inverse sigmoidal (α < 1, β < 1), concave (α ≤ 1, β > 1) or convex (α > 1, β ≤ 1). Furthermore, values for shape parameters could be set based on priors, i.e. $\alpha_{i,x}\;=\;f\left(\pi_{i,x}\right)$ and $\beta_{i,x}\;=\;f\left(\pi_{i,x}\right)$, as well as separately for positive and negative conditional dependencies. Even with this extra flexibility, notice that the SUM model in eqn 3 still assumes that species identity does not matter.

An intermediate option that retains some notion of species identity but is less empirically demanding than the FULL model involves assigning a heuristic ‘strength’ to each biotic interaction. For example, we could propose three classifications: ‘weak’ = 1, ‘medium’ = 2 and ‘strong’ = 3. Each biotic interaction is assigned one of the classifications, then the associated numbers are summed to determine new values for $n_{+,i}$, $n_{-,i}$, $n_{+,i}^{\max}$ and $n_{-,i}^{\max}$. These values, through $g_{+,i}$ and $g_{-,i}$, can be used in eqn 3 as a modified SUM model. It would be straightforward to extend this heuristic‐based approach to model further biological realism, including systematic differences between different types of biotic interaction (e.g. trophic, seed dispersal, pollination or parasitism). Of course, there are many other ways and potential rules for filling state table entries, and with small communities it may even be possible to empirically parameterise entries for all species.

## Application to Species from a California Grassland Community

### Species pool

The species pool for modelling was defined by plant species detection in a California (USA) grassland over 18 years of field research, plus the most abundant shared consumer of these species at the study site, a generalist grasshopper. Since 1999, occurrences and abundances of plant species have been monitored in a grassland at the Angelo Coast Range Reserve in Mendocino County ($39^{\circ }44^{\prime }17.7^{\prime \prime }$ N, $123^{\circ }37^{\prime }48.4^{\prime \prime }$ W). We downloaded presence records for the 53 plant species detected over this study period and the generalist grasshopper *Melanoplus devastator* from the Global Biodiversity Information Facility (http://GBIF.org). We used only records that had a known year and known basis (e.g. human observation or herbarium specimen). To align with the climate data (see below), we used records from the years 1970–2000 and, for each species, classified as ‘present’ any 800 m × 800 m grid cell that contained one or more occurrence records. To make our demonstration of the method more computationally tractable, and because many of the presence records were in California and the surrounding region, we restricted our analysis to the western USA ($32^{\circ }$ N to $49.1^{\circ }$ N and $125^{\circ }$ W to $115^{\circ }$ W). Across this geographical extent, 37 species had fewer than 30 presence records, so we focused on the 14 species with the most presence records (36–94 occurrences; Table 1) when validating model performance and assessing range changes because model performance is better with more records (Pearson *et al*. 2007; names and functional groups of all 54 species are listed in Table S1 in *Supporting Information*).

**Table 1:** List of the 14 focal species with the most presence records in the western USA extent

Species ID	Name	Functional group	Number of presence records
1	*Achillea millefolium*	Perennial forb	94
2	*Aira caryophyllea*	Annual grass	45
7	*Bromus carinatus*	Perennial grass	77
9	*Bromus hordeaceus*	Annual grass	39
11	*Bromus tectorum*	Annual grass	67
19	*Danthonia californica*	Perennial grass	38
22	*Draba verna*	Spring forb	48
23	*Elymus glaucus*	Perennial grass	80
25	*Epilobium brachycarpum*	Summer forb	75
36	*Madia gracilis*	Summer forb	53
43	*Ranunculus occidentalis*	Perennial forb	55
44	*Rumex acetosella*	Perennial forb	44
52	*Trifolium microcephalum*	Nitrogen‐fixing forb	49
54	*Vulpia myuros*	Annual grass	36

Species IDs were assigned in alphabetical order and a complete list of all 54 species are in Table S1.

### Characterising community‐level interactions

We characterised community‐level interactions from a series of transplant and competitor‐removal experiments (Thomsen *et al*. 2006; Suttle & Thomsen 2007), consumer addition and removal experiments (Suttle *et al*. 2007), and monitoring of species’ composition, production, and population abundances in replicate $70\;m^{2}$ plots over 16 years under ambient climate variability and experimental forcings in rainy season intensity and duration (Suttle *et al*. 2007; Sullivan *et al*. 2016).

A major point of context for understanding community‐level interactions in the study system is the phenological diversity of plant species in California grasslands. Any consideration of interspecific interactions among plant species must account for the different seasonal periods in which species are active. To this end, we categorised the 53 plant species into eight functional groups according to phenology and basic life history traits, then used these categories to summarise expected conditional dependencies for plant‐plant interactions, along with herbivore plant interactions between the generalist grasshopper *Melanoplus devastator* (species ID 37) and plants in each of the eight functional groups (Table 2).

**Table 2:** Community‐level interactions for the modelled species pool based on 16 years of experiments and field research in a northern California grassland

	WF	SpF	SuF	NF	PF	AG	PG	Bulb	MD
WF	•	+	+			−			
SpF		•		−					
SuF			•						
NF		−		•		+			
PF					•				
AG	−	−	−			•			
PG			−				•		
Bulb								•	
MD		−	−			−			•

Plant species are categorised into eight functional groups according to phenology and basic life history traits: winter forbs (WF); spring forbs (SpF); summer forbs (SuF); nitrogen‐fixing forbs (NF); perennial forbs (PF); annual grasses (AG); perennial grasses (PG); and bulbs (Bulb). Also included are interactions for the generalist grasshopper *Melanoplus devastator* (MD, species ID 37). Positive (+) and negative (−) interaction signs represent the effect of row species on column species.

Annual grasses are the most notable competitors in California grasslands, but their early phenology means their activity only overlaps with a subset of annual forbs. Annual forbs can be divided into winter, spring and summer forbs according to their differing phenologies, with direct competition mostly between winter forbs and annual grasses. Annual grasses also have competitive effects on spring and summer forbs, but this form of competition mainly comes from annual grass litter inhibiting germination and growth of the later‐season forbs, so there are no reciprocal effects of spring and summer forbs on annual grasses. Unlike the persistent thatch of annual grasses, winter forb tissues break down rapidly as plants senesce, so these species provide a favourable germination and growth environment for spring and summer forbs. An additional litter‐based effect extends across plant growing seasons: nitrogen‐fixing forbs in one growing season fuel increased annual grass production in the next season due to a fertilisation effect from the breakdown of nitrogen‐rich tissues during the winter rainy season when annual grasses are active.

Perennial grasses, perennial forbs and perennial bulbs are also present in the system. Individuals of these species are relatively tolerant of competition from surrounding annuals, and their populations fluctuate much less than annual species because their reproductive strategy relies more on the survival of existing individuals and less on the recruitment of new individuals relative to annual species. The main competitive effect of perennial plants in the study system occurs in the summer, when perennial grasses are active alongside summer forbs and there is competition for limited water resources. The generalist grasshopper *Melanoplus devastator* is an important consumer of spring forbs, summer forbs, and annual grasses; winter forbs senesce early enough to precede grasshoppers in the system, and perennial plants are highly tolerant of herbivory.

Further details about community‐level interactions were revealed from environmental change experiments that extended the rainy season from the usual January–March period. Since 2001, a random selection of the $70\;m^{2}$ plots at the study grassland have been experimentally subject to 14–16 mm of additional rainfall delivered every 3 days during April, May and June (Suttle *et al*. 2007; Sullivan *et al*. 2016). Two plant species in the 53‐species record for the study system occur only in plots with the extended rainy season treatment, and appeared only after many years of repeated extensions (*Stachys ajugoides*, ID 47, after 7 years and *Cirsuim occidentale*, ID 15, after 9 years). Two other species (*Aira caryophyllea*, ID 2, and *Epilobium brachycarpum*, ID 25), common throughout the grassland, have entirely dropped out of plots with the extended rainy season treatment. This dynamic could signal heightened competition among the four species, or it could represent a negative corollary to shared habitat suitability, whereby the directional environmental forcing creates mutually exclusive habitat suitability between the more mesic‐associated *Stachys ajugoides* and *Cirsuim occidentale* and the more xeric‐associated *Aira caryophyllea* and *Epilobium brachycarpum*.

### Environmental data

We selected climate variables developed for an earlier, unrelated study (Pearson *et al*. 2014) and freely available online (https://doi.org/10.7917/D7WD3XH5). Briefly, an ensemble of five atmosphere‐ocean general circulation models was used to generate climate anomalies for the years 2010 and 2050 based on a reference greenhouse gas emission scenario that assumes no substantive intervention to curb emissions (a pathway to stabilising atmospheric concentration at 750 ppmv, Wigley *et al*. 1996, which is similar to IPCC RCP 6.0, IPCC Fifth Assessment Report 2014). Climate anomalies were downscaled to an ecologically relevant spatial resolution of ∼ 800 m × 800 m and added to a high‐resolution baseline observed climatology (PRISM 1971–2000 normals; http://prism.oregonstate.edu). We selected seven bioclimate variables for use in SDMs based on their relevance to the California grassland community: maximum temperature of the warmest month; minimum temperature of the coldest month; annual precipitation; precipitation of the driest quarter; mean temperature of the wettest quarter; temperature seasonality (standard deviation × 100); and precipitation seasonality (coefficient of variation). California experiences a Mediterranean‐type climate with hot, dry summers and cool, wet winters; prolonged annual drought is typical, so the distribution of temperature and rainfall has a large influence on the grassland ecosystem (Suttle *et al*. 2007; Sullivan *et al*. 2016).

### Species distribution models

We generated SDMs using the Maxent method (Phillips *et al*. 2006), which performs well compared to other types of distribution model (Elith *et al*. 2006). Although many different methods exist for estimating the geographical distribution of species (Elith *et al*. 2006), and multiple methods are often used to compare SDMs and find agreement among models (Araújo & New 2007; Thuiller *et al*. 2009), we used only one method as our goal was to illustrate the use of BNs for modelling biotic interactions. Our approach can be implemented with other types of SDM by following a similar workflow.

Maxent uses presence records for a species and associated environmental data to determine favourable habitat conditions, which are then combined with environmental data at other locations to estimate a geographical distribution for the species (Elith *et al*. 2011). We implemented Maxent using the BIOMOD platform in R (Thuiller *et al*. 2009). Models were calibrated for each species separately using the baseline climatology and then projected for years 2010 and 2050. We used Maxent's logistic output, which is an approximation of the species’ probability of occurrence and can be interpreted as the habitat suitability for each species in each grid cell (Elith *et al*. 2011). Habitat suitability values were transformed to a binary (1 = ‘present’ and 0 = ‘absent’) species geographical range by selecting a threshold value above which the species is assumed to be able to exist and below which it cannot exist. We considered two ways of selecting an appropriate threshold: (1) the largest habitat suitability value (and therefore smallest area) that resulted in all presence records being included in the estimated species range; and (2) the habitat suitability value that maximised the true skill statistic (TSS; Allouche *et al*. 2006), which is equivalent to maximising the sum of model specificity and sensitivity, known as maxSSS (Liu *et al*. 2013). Species ranges for 2010 represent the ‘present day’ and those for 2050 represent predictions under environmental change.

### A Bayesian network for the California grassland community

We specified the graphical structure of the BN using a strategy that is similar to an optimisation process but less computationally intensive, and for simplicity used the OR model (eqn 2) to fill state table entries. We determined a DAG in five steps: (1) for each of the 14 focal species, we computed the AUC when applying a BN with one positive conditional dependency involving the focal species as the child node and, in turn, each of the other 53 species as the parent node; (2) we repeated the first step but with negative conditional dependencies; (3) we repeated the first and second steps for a total of 20 different training and test partitions; (4) we averaged AUC scores over the 20 data partitions separately for each focal species; and (5) we constructed an initial directed graph that included all positive and negative conditional dependencies that consistently increased AUC, then pruned conditional dependencies using a greedy heuristic (Eades *et al*. 1993) to ensure that the final graph was acyclic. The resulting DAG is not expected to maximise AUC and there are likely other topologies which result in better AUC scores. It is also likely to omit many recognised biotic interactions, but their inclusion would be unlikely to affect subsequent analysis.

**Figure 2:**
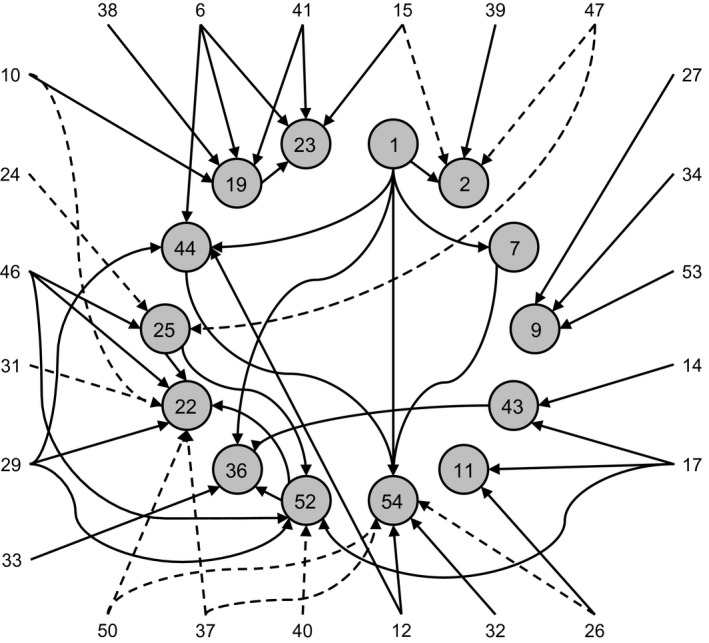
Bayesian network for the California grassland community. The 14 focal species with the most presence records across the western USA are shown as circles arranged in a central ring, and 23 less‐common species are shown as a square border (names and functional groups for the focal species are listed in Table 1, and in Table S1 for the other species). Positive conditional dependencies are illustrated by solid arrows (40 in total) and negative conditional dependencies by dashed arrows (12 in total). By construction, there are no conditional dependencies among non‐focal species. Notice there are multiple conditional dependencies per focal species that sometimes form chains of influence with multiple levels. We used the OR model (eqn 2) for filling state table entries with this Bayesian network.

The BN we obtained exhibits a complex topology with multiple conditional dependencies per focal species that sometimes form chains of influence (Fig. 2). Not counting the 14 focal species, 23 of the remaining 40 species were included in the BN as parent nodes (they were also all root nodes, by construction). The BN contained a total of 12 negative and 40 positive and conditional dependencies.

Of the twelve negative conditional dependencies identified by AUC optimisation, nine match expected community‐level interactions. Negative conditional dependencies between the generalist grasshopper *Melanoplus devastator* (species ID 37) and the spring forb *Draba verna* (ID 22) and the annual grass *Vulpia myuros* (ID 54) align with herbivore‐resource interactions in the study system, and the remaining seven negative conditional dependencies align well with expectations of competition. Four of these negative conditional dependencies may also represent a negative corollary to shared habitat suitability, with paired species requiring mutually exclusive sets of environmental conditions. AUC optimisation picked up negative conditional dependencies between the species that occur only in plots watered through spring and into summer (*Stachys ajugoides* and *Cirsuim occidentale*, ID 47 and ID 15, respectively) and the two species that do not occur at all in these plots (*Aira caryophyllea* and *Epilobium brachycarpum*, ID 2 and ID 25, respectively). Only two of the twelve identified negative conditional dependencies contradict community‐level understanding: those between the summer forb *Eremocarpus setigerus* (ID 26) and the annual grass *Vulpia myuros* (ID 54), and the nitrogen‐fixing forb *Trifolium albopurpureum* (ID 50) and *Vulpia myuros*. We do not have clear community‐level expectations for species in the same functional group based on research at the study site. For this reason, it is unclear how to interpret the final negative conditional dependency between the two spring forbs *Hemizonia congesta* (ID 31) and *Draba verna* (ID 22).

Of the 40 positive conditional dependencies, 32 appear to reflect shared habitat suitability, at least according to our definition of expected biotic interactions. The preponderance of positive compared to negative conditional dependencies is expected given the model's sensitivity to shared habitat suitability. As mentioned above, shared habitat suitability is not a biotic interaction between two species, but, as we show below, using positive conditional dependencies in this way still has predictive value. Six positive conditional dependencies match community‐level interactions, including the most conspicuous positive biotic interactions at the study site: facilitation of annual grasses by the breakdown of litter from nitrogen‐fixing forbs. Notably, the AUC optimisation procedure identified positive conditional dependencies between the most abundant nitrogen‐fixing forbs at the study site, *Lotus micranthus* (ID 34) and *Trifolium wildenovii* (ID 53), and the most abundant annual grass, *Bromus hordeaceus* (ID 9). In addition, there are positive conditional dependencies between the most abundant winter forb at the study site, the bedstraw species *Sherardia arvensis* (ID 46), and three later‐season forb species (IDs 22, 25 and 52), and also between the winter forb *Leptosiphon bicolor* (ID 33) and the summer forb *Madia gracilis* (ID 36). These results match interactions observed in the community in which winter forbs create favourable spaces for germination and establishment of spring and summer forbs, while annual grasses exclude these later‐season forbs. Two positive conditional dependencies contradict expected community‐level interactions: those between the annual grass *Gastridium ventricosum* (ID 29) and *Draba verna* (ID 22) and between the nitrogen‐fixing forb *Trifolium microcephalum* (ID 52) and *Draba verna*.

### Validating predictions of present‐day species ranges made with and without biotic interactions

We used AUC to measure model performance for the 14 focal species, separating presence records for each species into training (75% of presence records) and test partitions (25% of presence records). We also measured model performance using TSS, which gave qualitatively similar results to those using AUC (see Figs S1 and S2). Because AUC scores depend on the particular separation of data into training and test partitions, we considered 20 random partitions for each species.

For Maxent models without biotic interactions, we measured performance using AUC scores involving only prior habitat suitability values. When biotic interactions are ignored AUC is simply a function of the priors for one focal species $i\;=\;i^{*}$ calculated from the test partition, i.e. ${\text{AUC}}_{i=i^{*}}\;=f\left(\pi_{i=i^{*},\;x}^{\prime }\right)$, where $\pi_{i,x}^{\prime }$ indicates a value associated with a test partition to distinguish it from a value calculated using all available data. Computing AUC scores is more complicated when including biotic interactions with posteriors. This is because habitat suitability values must also be computed for all other species in addition to the focal species, while at the same time respecting the need to maintain comparable training and test partitions to the case without biotic interactions. When including biotic interactions with posteriors, we first computed priors for all species using all available data, i.e. $\pi_{i,x}$. Then we calculated AUC using the same training and test partitions for each focal species as in the case without biotic interactions, but all other priors were calculated using all available data, i.e. ${\tilde{\text{AUC}}}_{i=i^{*}}\;=\;f\left(p_{i=i^{*},\;x}^{\prime }\right)\;=\;f\left(\pi_{i=i^{*},x}^{\prime },\;\pi_{i\neq i^{*},x}\right)$. This allowed us to isolate the effect of biotic interactions on AUC separately for each focal species, with paired ${\text{AUC}}_{i=i^{*}}$ and ${\tilde{\text{AUC}}}_{i=i^{*}}$ scores for each training and test partition. If $\Delta {\text{AUC}}_{i=i^{*}}\;=\;{\tilde{\text{AUC}}}_{i=i^{*}}\;-\;{\text{AUC}}_{i=i^{*}}$ is consistently greater than zero across multiple training‐test partitions, then the BN is valuable and including biotic interactions in SDMs improves estimates of species ranges.

**Figure 3:**
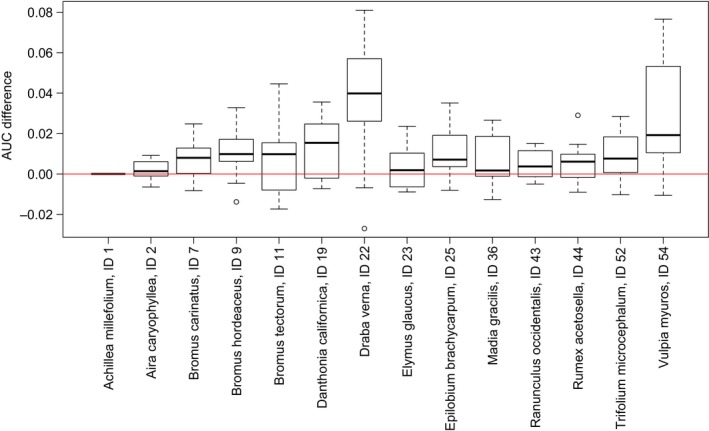
Increases and decreases in AUC scores for the 14 focal species when including biotic interactions in species distribution models (SDMs). For each focal species, we calculated the difference in the AUC score when using posteriors (output of an SDM with biotic interactions) and when using priors (output of an SDM without biotic interactions) for the same partition of data into training and test sets. Paired differences in AUC were calculated for 20 random partitions for each focal species. Box plots show interquartile range, median, minimum and maximum values of paired differences in AUC. If the difference in AUC is consistently greater than zero, then the BN is valuable and including biotic interactions in SDMs improves estimates of species geographical ranges.

We found that modelling biotic interactions with the BN for the California grassland community consistently improved AUC scores for 6 of the 14 focal species (Fig. 3, interquartile range completely above zero for species IDs 7, 9, 22, 25, 52 and 54). There were noticeable but less pronounced improvements in AUC for a further five species (interquartile range mainly above zero for IDs 11, 19, 36, 43 and 44). One species (*Achillea millefolium*, ID 1) had no conditional dependencies in the BN (it was a root node), so there was no difference between priors and posteriors and therefore AUC. (TSS scores improved for five species but worsened for one species, see Fig. S2.) AUC scores were already high for priors, which restricted the potential for increases once biotic interactions were included in models (eight focal species had median AUC scores above 0.8, see Fig. S3). Using all data in SDMs for 2010, we found that posterior habitat suitability values ($p_{i,x}$) were typically higher than prior values ($\pi_{i,x}$), which was unsurprising given the greater number of positive conditional dependencies in the BN. Species ranges were also typically larger with posteriors than priors, a result that was more pronounced with the inclusion threshold because the maxSSS threshold allows omission of some presence records and therefore results in smaller estimated species ranges.

### Comparing predictions of future impacts from climate change made with and without biotic interactions

After testing whether modelling biotic interactions using a BN improved model performance with 2010 data, we made predictions of species ranges in 2050 using projected climate variables in SDMs (projections suggest warmer and drier conditions in 2050 across the western USA; see Table S2). Estimating the impacts of climate change on species is one of the most common applications of SDMs, with many studies predicting high risk of extinctions over the coming decades (Thomas *et al*. 2004; Pearson *et al*. 2014; Urban 2015). Although it is well documented that such predictions remain highly uncertain (e.g. they fail to account for potentially rapid genetic adaptation and phenological mistiming; Pearson & Dawson 2003; Parmesan 2006), we aim here to demonstrate how incorporating biotic interactions refines predictions and moves us a step closer to being able to make reliable assessments of future impacts from climate change.

For each focal species, we assessed the predicted change in its geographical range between 2010 and 2050 first using a model without biotic interactions to serve as a reference point and then using a model with biotic interactions. We quantified change between 2010 and 2050 in three ways: (1) the change in average habitat suitability; (2) the change in binary range when the threshold habitat suitability was set to the largest value that resulted in all presence records being included in the species range for 2010; and (3) the change in binary range when the threshold habitat suitability was set to the maxSSS value for 2010 data. For both threshold‐based measures, we assumed that species dispersal was either possible (the predicted range in 2050 could extend beyond the range in 2010) or not (the predicted range in 2050 was limited to grid cells classed as ‘present’ in 2010) (Thomas *et al*. 2004). Predicted decreases in species ranges from this kind of assessment have been widely interpreted as indicating increased risk of extinction (Urban 2015). We provide a detailed description of how range changes were quantified in Appendix S2 and results in Appendix S3.

**Figure 4:**
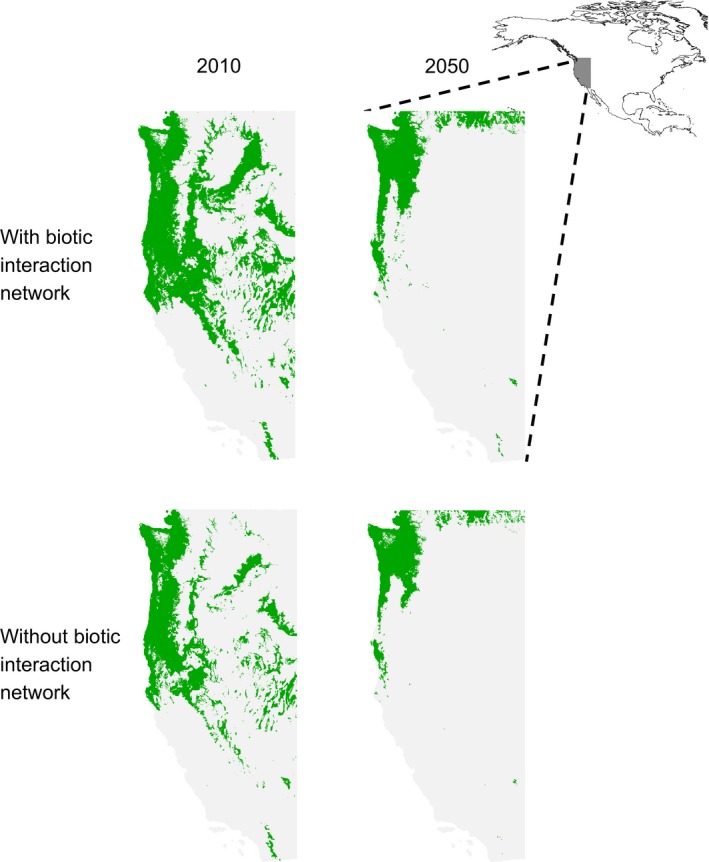
Predicted change in the geographical range of *Trifolium microcephalum* (nitrogen‐fixing forb, species ID 52) between 2010 (left) and 2050 (right) using species distribution models (SDMs) with (top) and without (bottom) biotic interactions and shared habitat suitability relationships. We considered SDM outputs for the western USA at a resolution of ∼ 800 m × 800 m grid cells. Habitat suitability values were transformed to a binary (green = ‘present’ and grey = ‘absent’) species range using the maxSSS threshold and allowing for dispersal. The two species ranges for 2010 represent the ‘present day’, and the two species ranges for 2050 are based on a greenhouse gas emission scenario that assumes no substantive intervention to curb emissions. Notice that both models predict a smaller geographical range in 2050, but the SDM with biotic interactions and shared habitat suitability relationships predicts a larger relative decrease in geographical range (values in Table S3). Differences between range maps are highlighted in Fig. S16.

In general, we found that ignoring biotic interactions led to larger predicted decreases in species ranges; however, there were also examples where ignoring biotic interactions led to smaller decreases. Results also varied by how change was quantified. For instance, the SDM without biotic interactions for *Danthonia californica* (ID 19) predicted a 30% decrease in range between 2010 and 2050 (using the maxSSS threshold with dispersal), whereas the model with biotic interactions predicted a smaller decrease of 11% (Table S3). By contrast, for *Trifolium microcephalum* (ID 52) the SDM with biotic interactions predicted a larger relative decrease in geographical range than the SDM without biotic interactions (65% with interactions vs. 59% without interactions; maxSSS threshold with dispersal; Table S3). However, note that the SDM with biotic interactions for *Trifolium microcephalum* predicted a larger geographical range than the model without biotic interactions in both 2010 and 2050 (Fig. 4), which highlights a limitation of quantifying impacts based only relative change (see Figs S4–S17 for geographical ranges for the other focal species). For one species, *Epilobium brachycarpum* (ID 25), including biotic interactions reversed the predicted response from a 16% increase in range to a 6% decrease (maxSSS threshold with dispersal; Table S3). Further research with a larger sample size of species across multiple taxonomic groups will be required to discern whether predicted impacts of climate change on biodiversity are generally more or less severe when biotic interactions are included in the models, or if there is no general trend. Nevertheless, our application here demonstrates that including biotic interactions is possible and has the potential to substantially affect results.

## Discussion

We have presented an approach that links macro‐scale species distributions and local‐scale community interactions by combining SDMs and BNs. The approach addresses a long‐standing problem in macroecology: that models of species distributions have focused on the abiotic constraints imposed on species distributions (the Grinnellian niche) and failed to take into account biotic interactions (the Eltonian niche). Improvements in predictions of present‐day species’ distributions when biotic interactions were included were relatively small but nevertheless consistent for our application to a Californian grassland (when SDM performance was measured using AUC). The fact that improvements in predictions were only small may be because ranges at large scales are determined principally by abiotic factors, in line with the Eltonian Noise Hypothesis; however, further research with more species in different regions will be required to robustly test this hypothesis. We have shown that including biotic interactions can substantially alter assessments of range changes under future environmental change, including the possibility that projections will reverse from an increasing range to a decreasing range. We therefore expect that linking SDMs and BNs will lead to improved estimates of species distributions across a wide range of applications, including conservation planning, invasive species management, and disease containment (Peterson *et al*. 2011).

### Specifying and validating Bayesian networks

We have described two general approaches to specifying the graphical structure of a BN: defining it based on community‐level knowledge or inferring it from macroecological occurrence data. Specifying a BN directly from local scale, community data is a promising avenue for future work, particularly because it enables true biotic interactions to be separated from dependencies that reflect shared habitats. However, the macroecological approach that we illustrate for the California case study also offers great potential, not only because it makes use of occurrence data sets that are more readily available, but also because it enables identification of interactions that should be included in models to improve predictive performance.

Using macroecological models to infer interactions can also enhance community‐level understanding because the effects of biotic interactions can vary across present and future modelled ranges. Even iconic examples of strongly interacting species show pronounced variation in interaction strength and importance at different locations within their ranges (Paine 1966; Dayton 1971; Menge *et al*. 2004). In addition to differences owing to local community composition, environmental variation across years within a single study system has revealed that important structuring interactions are a function of specific physical contexts rather than some kind of robust ecological truth (Power, 1990, Power *et al*. 2008). As such, the possibility for macroecological models to inform community‐level understanding can be just as useful as using community‐level knowledge to inform macroecological modelling.

Further work could explore the use of different BNs at different locations for the same community. For example, repeating local experimental manipulations over macroecological extents (the comparative‐experimental approach; Paine 2010) will prove informative for specifying different BNs. Different BNs spread across space could then be embedded within a metacommunity framework (Cazelles *et al*. 2016). BNs could also vary through time as biotic interactions are altered by, for instance, the introduction of an invasive alien species or by climate change (Blois *et al*. 2013), and experimental methods to quantify novel biotic interactions (Alexander *et al*. 2016) have potential to inform in this regard.

Note that the two general approaches to specifying a BN are complementary when it comes to validation (see Appendix S5). When predicting species ranges, a general way of assessing the value of a BN is by comparing the value of an objective function when an SDM includes the BN to when it does not, for example by comparing the AUC with posteriors to the AUC with priors, as we did in the California grassland case study. Similarly, different BNs can be compared using AUC to test the importance of specific groups of biotic interactions or modelling assumptions for a given system. This approach is particularly informative when a BN is specified using data from local observations or experimental manipulations.

When macroecological occurrence data are combined with an optimisation procedure, the resulting BN is by definition the ‘best’ model, so validation requires understanding whether the suggested interactions make biological sense (for a discussion of penalising for model complexity, see Appendix S5). As demonstrated here, the simplified representation of biotic interactions in a BN can be associated with ecological processes known to be important for the system, such as facilitation by nitrogen fixers on annual grasses and some forbs, and competition between annual grasses and other plant species.

### Future prospects

We used the Maxent method for SDMs, but BNs can be easily combined with other machine‐learning (e.g. Boosted Regression Trees), regression‐based (e.g. generalised additive models) or envelope‐based methods (e.g. Bioclim) (Elith *et al*. 2006). The most useful and mathematically satisfying approach would involve SDMs that output actual probabilities of species occurrence across a geographical extent (as opposed to scores for relative habitat suitability). Because correlative SDMs such as Maxent characterise suitable abiotic environments based on observed ranges, which reflect biotic as well as abiotic controls, these models implicitly incorporate a measure of biotic interactions. By contrast, the approach we propose here enables biotic interactions to be modelled explicitly, enabling knock‐on impacts across a community to be captured. BNs could also be combined with mechanistic distribution models, which utilise information on the physiological constraints that abiotic variables place on species independent of biotic factors (Kearney & Porter 2009). And because mechanistic distribution models do not implicitly incorporate biotic interactions, we expect to see greater predictive improvement when biotic interactions are added explicitly using BNs.

As expected, because conditional dependencies include the effects of shared habitat preferences as well as more direct facilitation, we found more positive than negative conditional dependencies in the BN for the California grassland community. Future analyses of the effects of spatial resolution are likely to reveal increased model performance when biotic interactions are included at finer spatial resolutions. Despite a relatively small grid‐cell size for macroecological studies (∼ 800 m × 800 m), even this resolution may not be fine enough to identify competitive exclusion and other negative conditional dependencies, which are more likely to be observed at finer resolutions than positive associations among species (Araújo & Rozenfeld 2014; Thuiller *et al*. 2015). We therefore expect that as spatial resolution increases our approach will identify more biotic interactions, especially negative ones, and the effects of biotic interactions on predictions will become stronger.

Our approach will become more widely applicable as coverage of occurrence (and absence) records increases and new data sets on interactions among species in a community are assembled. Because the time required to solve a BN (transforming priors to posteriors) grows linearly with spatial scale and sub‐linearly with the number of species, adding this step to an existing workflow requires little computational overhead, even for large communities. Clearly, the number and pattern of biotic interactions will vary among ecological communities, and so will the effect of including biotic interactions in SDMs. It has been suggested, for example, that biotic interactions may have a greater effect on species at higher trophic levels (Ockendon *et al*. 2014). We presented three relatively simple sets of rules for modelling state table entries (eqns (1), (2), (3)). Such rules can and should be tailored for different types of interaction. However, it is imperative that laboratory and field experiments are not only used to parameterise rules, but also to justify the application of more complex sets of rules.

The approach we present offers two stimulating prospects: first as a way of testing knowledge about biotic interactions at large spatial scales, and second as a way of learning about biotic interactions from biogeographical data. Ecology rests on interactions between species, but the degree to which local‐scale interactions are important for predicting large‐scale distributions remains unclear. We have presented a way of incorporating biotic interactions in models of species distributions, which we hope will prove valuable for testing hypotheses that link macroecology and community ecology.

## Authorship

PPAS was responsible for research planning, analysis and writing the first draft; PS for software development and analysis; KBS for analysis and interpretation of modelling results; and RGP for research planning and analysis. All authors discussed the results and edited the manuscript.
