# Supplementary material for: Linking macroecology and community ecology: refining predictions of species distributions using biotic interaction networks
Source: Ecol Lett. 2017 Apr 21;20(6):693–707. doi: 10.1111/ele.12770 (PMC5485222; doi:10.1111/ele.12770)
Supplement: Supplementary file 1 [file ELE-20-693-s001.pdf]

SUPPORTING INFORMATION

Linking macroecology and community ecology: Refining predictions of species distributions using biotic interaction networks

Phillip P.A. Staniczenko[1,2,3,†], Prabu Sivasubramaniam[3,4,‡], K. Blake Suttle[5,§] & Richard G. Pearson[3,*]

[1]National Socio-Environmental Synthesis Center (SESYNC), Annapolis, MD, USA
[2]Department of Biology, University of Maryland, College Park, Maryland, MD, USA
[3]Centre for Biodiversity and Environment Research, University College London, London, UK
[4]School of Biological Sciences, Institute of Quantitative Biology, Biochemistry and Biotechnology, University of Edinburgh, Edinburgh, UK (present address)
[5]Department of Ecology and Evolutionary Biology, University of California, Santa Cruz, CA, USA
[†]pstaniczenko@sesync.org [‡]p.siva@ed.ac.uk [§]kbsuttle@ucsc.edu
[*]Correspondence should be addressed to richard.pearson@ucl.ac.uk
Centre for Biodiversity and Environment Research, University College London,
Gower Street, London, WC1E 6BT, United Kingdom

This document contains five appendices, three tables and seventeen figures

Contents

Appendix S1. Example R code for solving a Bayesian network. Also as file: `ELEstaniczenkoSA1.R`

Appendix S2. Additional methods and results for comparing predictions of future impacts from climate change made with and without biotic interactions.

Appendix S3. Excel file with calculations of changes in average habitat suitability for the 14 focal species, and corresponding changes in geographical range using the inclusion threshold and maxSSS threshold. File name: `ELEstaniczenkoSA3.xlsx`

Appendix S4. An extended discussion of validating Bayesian networks.

Appendix S5. An extended discussion of penalising for model complexity.

Table S1. Species list and number of presence records in the western USA extent.

Table S2. Climate variables at the Angelo Coast Range Reserve, California (39° 44′ 17.7″ N, 123° 37′ 48.4″ W).

Table S3. Changes in average habitat suitability and geographical range between 2010 and 2050 for the 14 focal species using models with and without biotic interactions.

Figure S1. TSS scores for the 14 focal species without biotic interactions in SDMs.

Figure S2. Increases and decreases in TSS scores for the 14 focal species when including biotic interactions in SDMs.

Figure S3. AUC scores for the 14 focal species without biotic interactions in SDMs.

Figure S4. Predicted change in the geographical range of *Achillea millefolium* (perennial forb, species ID 1) between 2010 and 2050 using SDMs with and without biotic interactions and shared habitat suitability relationships.

Figure S5. Predicted change in the geographical range of *Aira caryophyllea* (annual grass, species ID 2) between 2010 and 2050 using SDMs with and without biotic interactions and shared habitat suitability relationships.

Figure S6. Predicted change in the geographical range of *Bromus carinatus* (annual grass, species ID 7) between 2010 and 2050 using SDMs with and without biotic interactions and shared habitat suitability relationships.

Figure S7. Predicted change in the geographical range of *Bromus hordeaceus* (annual grass, species ID 9) between 2010 and 2050 using SDMs with and without biotic interactions and shared habitat suitability relationships.

Figure S8. Predicted change in the geographical range of *Bromus tectorum* (annual grass, species ID 11) between 2010 and 2050 using SDMs with and without biotic interactions and shared habitat suitability relationships.

Figure S9. Predicted change in the geographical range of *Danthonia californica* (perennial grass, species ID 19) between 2010 and 2050 using SDMs with and without biotic interactions and shared habitat suitability relationships.

Figure S10. Predicted change in the geographical range of *Draba verna* (spring forb, species ID 22) between 2010 and 2050 using SDMs with and without biotic interactions and shared habitat suitability relationships.

Figure S11. Predicted change in the geographical range of *Elymus glaucus* (perennial grass, species ID 23) between 2010 and 2050 using SDMs with and without biotic interactions and shared habitat suitability relationships.

Figure S12. Predicted change in the geographical range of *Epilobium brachycarpum* (summer forb, species ID 25) between 2010 and 2050 using SDMs with and without biotic interactions and shared habitat suitability relationships.

Figure S13. Predicted change in the geographical range of *Madia gracilis* (summer forb, species ID 36) between 2010 and 2050 using SDMs with and without biotic interactions and shared habitat suitability relationships.

Figure S14. Predicted change in the geographical range of *Ranunculus occidentalis* (perennial forb, species ID 43) between 2010 and 2050 using SDMs with and without biotic interactions and shared habitat suitability relationships.

Figure S15. Predicted change in the geographical range of *Rumex acetosella* (perennial forb, species ID 44) between 2010 and 2050 using SDMs with and without biotic interactions and shared habitat suitability relationships.

Figure S16. Predicted change in the geographical range of *Trifolium microcephalum* (nitrogen-fixing forb, species ID 52) between 2010 and 2050 using SDMs with and without biotic interactions and shared habitat suitability relationships.

Figure S17. Predicted change in the geographical range of *Vulpia myuros* (annual grass, species ID 54) between 2010 and 2050 using SDMs with and without biotic interactions and shared habitat suitability relationships.

Appendix S1. Example R code for solving a Bayesian network.

This code is adapted from Eklöf *et al.* 2013, with thanks to the authors.

Note that some dependencies of the required R package "gRain" are no longer hosted on CRAN. However, these dependencies can be obtained from the "bioconductor" package.

To install the dependencies, in R, type:

```
source(‘‘http://bioconductor.org/biocLite.R’’)
biocLite()
biocLite(pkgs=c(‘‘RBGL’’))
```

References

Eklöf, A., Tang, S. & Allesina, S. (2013). Secondary extinctions in food webs: a Bayesian network approach. *Methods Ecol. Evol.*, 4, 760–770.


```
# Example R code for solving a Bayesian network
# Consider the Bayesian network with three species:
# A -> C <-/- B
# where the presence of C is positively affected by A and negatively by B
# At a particular location, assume the species have prior probabilities of presence
# A: 0.7; B: 0.2; and C: 0.4

# Load the required R package for solving Bayesian networks
require(gRain)

# Define the state table (conditional probability table) for A
A <- cptable(~A, values=c(0.7,0.3), levels=c(‘present’, ‘absent’))

# Define the state table (conditional probability table) for B
B <- cptable(~B, values=c(0.2,0.8), levels=c(‘present’, ‘absent’))
```

```
# Define the state table (conditional probability table) for C
# The order is (A=present,B=present) (absent,present) (present,absent) (absent,absent)
C <- cptable(∼C|A+B, values=c(0.4,0.6, 0,1, 0.8,0.2, 0.4,0.6), levels=c(‘present’,
‘absent’))
# Compile state tables (conditional probability tables)
plist <- compileCPT(list(A, B, C))
# Build the Bayesian network
BN <- grain(plist)
# Solve the Bayesian network
posteriors <- querygrain(BN)
# Display results
print(posteriors)
# The posteriors should be 0.7, 0.2 and 0.6 for species A, B and C, respectively
# (as explained in Box 1 in the main text).
```

Appendix S2. Additional methods and results for comparing predictions of future impacts from climate change made with and without biotic interactions.

Formally, we quantified the change in habitat suitability for focal species $i = i^*$ as

$$R^{\text{HS}}_{i=i^*} = \frac{\sum\limits_{x=1}^{x=X} \pi^{2050}_{x,i=i^*} - \sum\limits_{x=1}^{x=X} \pi^{2010}_{x,i=i^*}}{\sum\limits_{x=1}^{x=X} \pi^{2010}_{x,i=i^*}} \tag{S1}$$

for models without biotic interactions, and as

$$\widetilde{R}^{\text{HS}}_{i=i^*} = \frac{\sum\limits_{x=1}^{x=X} p^{2050}_{x,i=i^*} - \sum\limits_{x=1}^{x=X} p^{2010}_{x,i=i^*}}{\sum\limits_{x=1}^{x=X} p^{2010}_{x,i=i^*}} \tag{S2}$$

for models with biotic interactions, where X is the total number of grid cells in the western USA extent. When habitat suitability values were thresholded (to the largest value that resulted in all presence records being included in the geographical range for 2010 and to the maxSSS value for 2010 data), we calculated changes in geographical range as

$$R^{\text{Threshold}}_{i=i^*} = \frac{\text{Area}^{2050}_{i=i^*} - \text{Area}^{2010}_{i=i^*}}{\text{Area}^{2010}_{i=i^*}} \tag{S3}$$

for models without biotic interactions, and as

$$\widetilde{R}^{\text{Threshold}}_{i=i^*} = \frac{\widetilde{\text{Area}}^{2050}_{i=i^*} - \widetilde{\text{Area}}^{2010}_{i=i^*}}{\widetilde{\text{Area}}^{2010}_{i=i^*}} \tag{S4}$$

for models with biotic interactions, where $\text{Area}_{i=i^*}^{\text{Year}}$ is the sum of grid cells classed as "present" across the western USA extent using priors, and similarly with $\widetilde{\text{Area}}_{i=i^*}^{\text{Year}}$ for posteriors. Although modelling dispersal distances for each species would result in more realistic predictions (Urban *et al.* 2013), to demonstrate the methodology for integrating biotic interactions we here repeated the calculations both with full dispersal and without any dispersal.

Larger positive values of $R_{i=i^*}^{\text{HS}}$, $\widetilde{R}_{i=i^*}^{\text{HS}}$, $R_{i=i^*}^{\text{Threshold}}$ and $\widetilde{R}_{i=i^*}^{\text{Threshold}}$ are suggestive of lower extinction risk for the focal species because average habitat suitability or geographical range is predicted to increase between 2010 and 2050; conversely, larger negative values are suggestive of higher extinction risk[1]. We determined the effect of biotic interactions on predicted changes between 2010 and 2050 by comparing the same measure using priors and posteriors. For example, if $\Delta R^{\text{HS}} = \widetilde{R}_{i=i^*}^{\text{HS}} - R_{i=i^*}^{\text{HS}} < 0$ then the model with biotic interactions predicts either a smaller increase or larger decrease in average habitat suitability than the comparable model without biotic interactions. But if $\Delta R^{\text{HS}} = \widetilde{R}_{i=i^*}^{\text{HS}} - R_{i=i^*}^{\text{HS}} > 0$ then the model with biotic interactions predicts either a larger increase or smaller decrease in average habitat suitability than the comparable model without biotic interactions. A similar reasoning holds for the threshold-based measures: if $\Delta R^{\text{Threshold}} = \widetilde{R}_{i=i^*}^{\text{Threshold}} - R_{i=i^*}^{\text{Threshold}} > 0$ then the model with biotic interactions predicts either a larger increase or smaller decrease in the species range (area) than the comparable model without biotic interactions.

When considering the change in average habitat suitability between 2010 and 2050,

[1] We used proportional changes in average habitat suitability and geographical range rather than absolute changes to minimise any bias due to systematic differences in values between priors and posteriors. Results were qualitatively similar with both approaches except for changes in average habitat suitability, in which case, absolute changes were consistently larger with posteriors than priors due to larger predicted values in 2010, see Appendix S3.

results for priors ($R^{\mathrm{HS}}_{i=i^*}$, Eqn S1) were typically lower than for posteriors ($\widetilde{R}^{\mathrm{HS}}_{i=i^*}$, Eqn S2). Across the 14 focal species, $\Delta R^{\mathrm{HS}} > 0$ for 9 species (10.1±18.3 percentage points, mean ± SD; min: 0.6; max: 58.6) and $\Delta R^{\mathrm{HS}} < 0$ for 4 species (11.2 ± 7.8 percentage points; min: 4.5; max: 22.0). (Recall that species ID 1 had no conditional dependencies in the BN, so there cannot be any difference between $\widetilde{R}^{\mathrm{HS}}_{i=i^*}$ and $R^{\mathrm{HS}}_{i=i^*}$ for this species.) There was qualitative agreement with results for the inclusion threshold-based measure (Eqns S3 and S4). With dispersal, $\Delta R^{\mathrm{Threshold}} > 0$ for 8 species (5.3 ± 5.1 percentage points; min: 1.6; max: 16.5) and $\Delta R^{\mathrm{Threshold}} < 0$ for 5 species (2.1 ± 2.2 percentage points; min: 0.2; max: 4.7). Without dispersal, $\Delta R^{\mathrm{Threshold}} > 0$ for 9 species (5.4 ± 5.9 percentage points; min: 1.1; max: 20.2) and $\Delta R^{\mathrm{Threshold}} < 0$ for 4 species (0.6 ± 0.3 percentage points; min: 0.4; max: 1.1). Results were less clear-cut using the maxSSS threshold. With dispersal, $\Delta R^{\mathrm{Threshold}} > 0$ for 6 species (6.2 ± 6.6 percentage points; min: 0.9; max: 18.2) and $\Delta R^{\mathrm{Threshold}} < 0$ for 7 species (11.0 ± 12.5 percentage points; min: 1.5; max: 34.9). Without dispersal, $\Delta R^{\mathrm{Threshold}} > 0$ for 6 species (6.2 ± 6.3 percentage points; min: 0.6; max: 16.6) and $\Delta R^{\mathrm{Threshold}} < 0$ for 7 species (4.2 ± 4.5 percentage points; min: 0.5; max: 13.6).

Changes in average habitat suitability and geographical range between 2010 and 2050 for the 14 focal species are in Table S3, and calculations using those values are in Appendix S3.

References

Urban, M.C., Zarnetske, P.L. & Skelly, D.K. (2013). Moving forward: dispersal and species interactions determine biotic responses to climate change. *Annals of the New York Academy of Sciences*, 1297, 44–60.

Appendix S3. Excel file with calculations of changes in average habitat suitability for the 14 focal species, and corresponding changes in geographical range using the inclusion threshold and maxSSS threshold.

File includes results for both proportional and absolute change in values, and is hosted at: `ELEstaniczenkoSA3.xlsx`

Appendix S4. An extended discussion of validating Bayesian networks.

Specifying Bayesian networks (BNs) based on community-level interaction data or inferred from macroecological occurrence data are two very different but complementary approaches that can be represented by a Venn diagram (see image, below). With either approach, the process of validation involves the intersection of the Venn diagram.

The Venn diagram contains two circles: (i) the set of biotic interactions defined from community-level data and (ii) the set of conditional dependencies inferred from macroecological data. In the main paper, the conditional dependences that we discussed as "validated" biotic interactions are contained in the intersection of the two circles. At the intersection, for the California grassland case study we identified nine negative and six positive conditional dependences that matched expectations based on community-level knowledge. The remainder of the interactions in circle (ii) comprise three negative conditional dependencies and thirty-four positive conditional dependencies, thirty-two of which could be explained by shared habitat suitability. The remainder of the interactions in circle (i) represent the set of biotic interactions that we did not but could have included in a BN based on community-level data. However, with our given macroecological occurrence data, these additional biotic interactions would likely have no effect on AUC scores or may even have a negative effect on AUC scores (due to the over-simplistic OR model we used, or inadequate number of occurrence records).

Through the Venn diagram, one can see that the correspondence between biotic interactions and conditional dependencies in a BN is centred on the process of validation. On

the one hand, community-level data can be used to define conditional dependencies in a BN, then their effect on model performance can be quantified using macroecological data. On the other hand, macroecological data can be used to infer conditional dependencies in a BN, then these inferred relationships can be contextualised using community-level knowledge. In practice, alternating between the two approaches is likely to be the most informative and powerful strategy. Community-level data can improve predictions of macroecological models; and macroecological data can inform understanding of community-level patterns and processes.

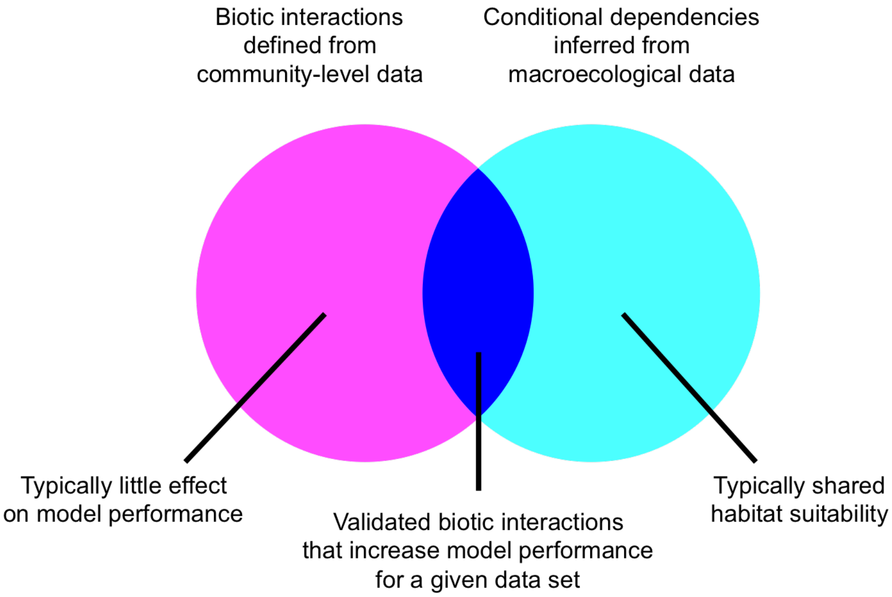

Venn diagram of the set of biotic interactions included in a Bayesian network based on community-level data and the set of conditional dependencies in a Bayesian network inferred from macroecological data.

Appendix S5. An extended discussion of penalising for model complexity.

In the main paper, we showed that consistent improvements in AUC were possible by modelling biotic interactions using Bayesian networks (BNs). Using macroecological occurrence data and an optimisation process, the BN we obtained for species from a California grassland community included a total of 52 conditional dependencies (12 negative and 40 positive). Nine negative and six positive conditional dependencies matched expected community-level interactions, thirty-two agreed with shared habitat suitability expectations, two negative and two positive contradicted community-level understanding, and there was no expectation for one conditional dependency.

If one is of the opinion that biotic interactions are fundamentally relevant for determining species ranges, then biotic interactions *should* be included in any model that attempts to predict species ranges. Given this viewpoint, any improvement in model performance due to including biotic interactions is beneficial, but also expected. Importantly, the approach we describe provides a way of quantifying whether or not it is *worth* including particular biotic interactions or sets of biotic interactions in species distribution models (for a given macroecological data set).

When BNs are inferred from macroecological occurrence data using an optimisation procedure, model performance is almost guaranteed to improve. This is why it is important to validate the biological relevance of inferred conditional dependencies using community-level knowledge.

Complexity penalisation is an important aspect of model selection, which is the process

of comparing models with potentially different numbers of parameters in order to find an overall best model. In general, the more complex a model—in terms of free parameters—the more likely it is to explain a given data set well. This inherent flexibility is the motivation behind penalising the raw performance of models by their complexity.

Accounting for BN model complexity is not straightforward. Consider the popular Akaike information criterion (AIC; Burnham & Anderson 2002), which penalises model likelihoods based on the number of parameters in a model. (Note that likelihood-based measures of model performance would typically require having actual probabilities of occurrence rather than habitat suitability values.) With BNs, it is not clear how parameters should be counted and used in AIC for a given BN. For example, the number of edges (conditional dependencies) is not appropriate because different BNs with the same number of edges but different topologies can have different numbers of possible states and therefore numbers of probabilities in state tables. The number of probabilities across all state tables is also not an appropriate number for penalising model complexity because all states are not equally likely.

One potentially useful approach to model selection comes from Information Theory and is called Normalized Maximum Likelihood (NML; Myung *et al.* 2006; Grünwald 2007). NML, like AIC, attempts to balance goodness-of-fit with model complexity. However, it does so using a very different approach, one that does not weight all model parameters by the same amount. This is important, because, as described above, the number of edges or distinct probabilities across state tables are not, in and of themselves, a good option for complexity penalisation.

NML quantifies how well a BN explains a particular data set compared to how well it explains all possible data sets of comparable size. An overly complex BN, often with many distinct probabilities, will fit many data sets well because of its flexibility, resulting in a small value for NML. An overly simple BN, such as one with no conditional dependencies, will fit all data sets by a similar amount, also resulting in a small value for NML. By contrast, a BN that fits only the observed data set well and all other data sets poorly would results in a large value for NML, and would be a good candidate for best model.

Although a model selection approach based on NML has been proposed for stochastic block models (Staniczenko *et al.* 2014)—which can be thought of as BNs without any conditional dependencies, i.e., all random variables are independent of one another—the added problem of accounting for conditional dependencies in BNs requires careful thought and further work. Nevertheless, NML and related information theoretic methods represent a promising general approach for model selection involving SDMs and BNs.

References

Burnham, K.P. & Anderson, D.R. (2002). *Model Selection and Multimodel Inference: A Practical Information-Theoretic Approach.* Springer, Second Edition.

Grünwald, P.D. (2007). *The Minimum Description Length Principle.* MIT Press.

Myung, J.I., Navarro, D.J. & Pitt, M.A. (2006). Model selection by normalized maximum likelihood. *J. Math. Psychol.*, 50, 167–179.

Staniczenko, P.P.A., Smith, M.J. & Allesina, S. (2014). Selecting food web models using normalized maximum likelihood. *Methods Ecol. Evol.*, 5, 551–562.

Table S1: List of species and numbers of presence records in the western USA extent. Highlighted rows are the 14 focal species with the most presence records.

Species ID	Name	Family	Functional group	Presence records
1	*Achillea millefolium*	Asteraceae	Perennial forb	94
2	*Aira caryophyllea*	Poaceae	Annual grass	45
3	*Aphanes occidentalis*	Rosaceae	Winter forb	18
4	*Avena barbata*	Poaceae	Annual grass	12
5	*Briza minor*	Poaceae	Annual grass	25
6	*Brodiaea elegans*	Asparagaceae	Bulb	16
7	*Bromus carinatus*	Poaceae	Perennial grass	77
8	*Bromus diandrus*	Poaceae	Annual grass	26
9	*Bromus hordeaceus*	Poaceae	Annual grass	39
10	*Bromus madritensis*	Poaceae	Annual grass	5
11	*Bromus tectorum*	Poaceae	Annual grass	67
12	*Cardamine oligosperma*	Brassicaceae	Spring forb	23
13	*Castilleja attenuata*	Orobanchaceae	Spring forb	12
14	*Cerastium glomeratum*	Caryophyllaceae	Spring forb	28
15	*Cirsium occidentale*	Asteraceae	Perennial forb	10
16	*Clarkia purpurea*	Onagraceae	Spring forb	27
17	*Convolvulus arvensis*	Convolvulaceae	Perennial forb	24
18	*Cynosurus echinatus*	Poaceae	Annual grass	32
19	*Danthonia californica*	Poaceae	Perennial grass	38
20	*Daucus pusillus*	Apiaceae	Summer forb	14
21	*Dichelostemma capitatum*	Asparagaceae	Bulb	13
22	*Draba verna*	Brassicaceae	Spring forb	48
23	*Elymus glaucus*	Poaceae	Perennial grass	80
24	*Elymus multisetus*	Poaceae	Perennial grass	6
25	*Epilobium brachycarpum*	Onagraceae	Summer forb	75
26	*Eremocarpus setigerus*	Euphorbiaceae	Summer forb	12

Species ID	Name	Family	Functional group	Presence records
27	*Eschscholzia californica*	Papaveraceae	Perennial forb	32
28	*Galium parisiense*	Rubiaceae	Spring forb	4
29	*Gastridium ventricosum*	Poaceae	Annual grass	2
30	*Geranium dissectum*	Geraniaceae	Spring forb	18
31	*Hemizonia congesta*	Asteraceae	Spring forb	5
32	*Hypochaeris glabra*	Asteraceae	Summer forb	13
33	*Leptosiphon bicolor*	Polemoniaceae	Winter forb	21
34	*Lotus micranthus*	Fabaceae	Nitrogen-fixing forb	9
35	*Lupinus bicolor*	Fabaceae	Nitrogen-fixing forb	31
36	*Madia gracilis*	Asteraceae	Summer forb	53
37	*Melanoplus devastator*	Acrididae	Generalist herbivore	7
38	*Micropus californicus*	Asteraceae	Winter forb	13
39	*Myosotis discolor*	Boraginaceae	Summer forb	29
40	*Navarretia divaricata*	Polemoniaceae	Spring forb	14
41	*Plagiobothrys nothofulvus*	Boraginaceae	Spring forb	11
42	*Platystemon californicus*	Papaveraceae	Spring forb	14
43	*Ranunculus occidentalis*	Ranunculaceae	Perennial forb	55
44	*Rumex acetosella*	Polygonaceae	Perennial forb	44
45	*Sanicula bipinnatifida*	Apiaceae	Perennial forb	21
46	*Sherardia arvensis*	Rubiaceae	Winter forb	23
47	*Stachys ajugoides*	Lamiaceae	Perennial forb	2
48	*Torilis arvensis*	Apiaceae	Summer forb	25
49	*Trichostema lanceolatum*	Lamiaceae	Summer forb	10
50	*Trifolium albopurpureum*	Fabaceae	Nitrogen-fixing forb	8
51	*Trifolium bifidum*	Fabaceae	Nitrogen-fixing forb	10
52	*Trifolium microcephalum*	Fabaceae	Nitrogen-fixing forb	49
53	*Trifolium willdenovii*	Fabaceae	Nitrogen-fixing forb	25
54	*Vulpia myuros*	Poaceae	Annual grass	36

Table S2: Climate variables at the Angelo Coast Range Reserve, California (39° 44′ 17.7″ N, 123° 37′ 48.4″ W).

Climate variable	2010	2050	Difference
Maximum temperature of the warmest month	27.56°C	29.25°C	1.69°C
Minimum temperature of the coldest month	2.10°C	3.23°C	1.13°C
Annual precipitation	2036.41mm	1939.02mm	-97.39mm
Precipitation of the driest quarter	36.47mm	29.57mm	-6.90mm
Mean temperature of the wettest quarter	8°C	10°C	2°C
Temperature seasonality (standard deviation $\times$ 100)	444	468	22
Precipitation seasonality (coefficient of variation)	84	83	-1

Table S3: Changes in average habitat suitability and geographical range between 2010 and 2050 for the 14 focal species using models with and without biotic interactions. Values were calculated using Eqns S1, S2, S3 and S4 from Appendix S2 with prior and post(erior) habitat suitability values. Larger positive values are suggestive of lower extinction risk whereas larger negative values are suggestive of higher extinction risk. Highlighted cells indicate when the model with biotic interactions predicts either a smaller increase or larger decrease in average habitat suitability or geographical range than the comparable model without biotic interactions.

	Habitat suitability		Inclusion threshold				maxSSS threshold			
			Dispersal		No dispersal		Dispersal		No dispersal	
Species ID	Prior	Post	Prior	Post	Prior	Post	Prior	Post	Prior	Post
1	-10.8%	-10.8%	-18.2%	-18.2%	-19.3%	-19.3%	-5.8%	-5.8%	-31.1%	-31.1%
2	-12.6%	-17.1%	-9.0%	-7.4%	-12.8%	-11.7%	-8.2%	-12.6%	-12.7%	-17.6%
7	-29.5%	-28.9%	-38.4%	-38.6%	-38.4%	-38.9%	-35.7%	-31.4%	-36.7%	-37.2%
9	-33.9%	-30.3%	-33.5%	-31.9%	-39.6%	-37.5%	-34.2%	-25.0%	-38.3%	-29.2%
11	-67.3%	-63.6%	-72.8%	-65.6%	-74.3%	-70.5%	-86.6%	-85.6%	-89.0%	-90.2%
19	-32.6%	-23.3%	-44.7%	-38.4%	-46.6%	-40.3%	-29.6%	-11.4%	-49.0%	-32.4%
22	-53.3%	-65.0%	-53.3%	-36.9%	-57.4%	-37.2%	-80.1%	-83.5%	-87.0%	-91.1%
23	-52.4%	-48.0%	-58.9%	-56.6%	-58.9%	-56.6%	-51.1%	-50.2%	-51.6%	-51.0%
25	12.6%	17.7%	-5.6%	-5.9%	-12.0%	-6.2%	15.8%	-5.9%	-18.9%	-32.6%
36	47.0%	25.0%	16.8%	12.4%	-5.0%	-5.4%	68.7%	33.7%	-27.7%	-19.8%
43	22.3%	80.9%	5.0%	4.0%	-12.1%	-13.1%	22.3%	17.6%	-15.8%	-16.7%
44	-70.6%	-67.7%	-68.9%	-67.3%	-69.2%	-67.8%	-81.2%	-77.6%	-81.5%	-79.2%
52	-60.3%	-57.3%	-67.5%	-62.3%	-69.9%	-64.3%	-58.7%	-64.7%	-74.2%	-78.4%
54	-23.3%	-29.9%	-24.8%	-29.5%	-31.3%	-31.7%	-31.1%	-32.6%	-34.9%	-34.3%

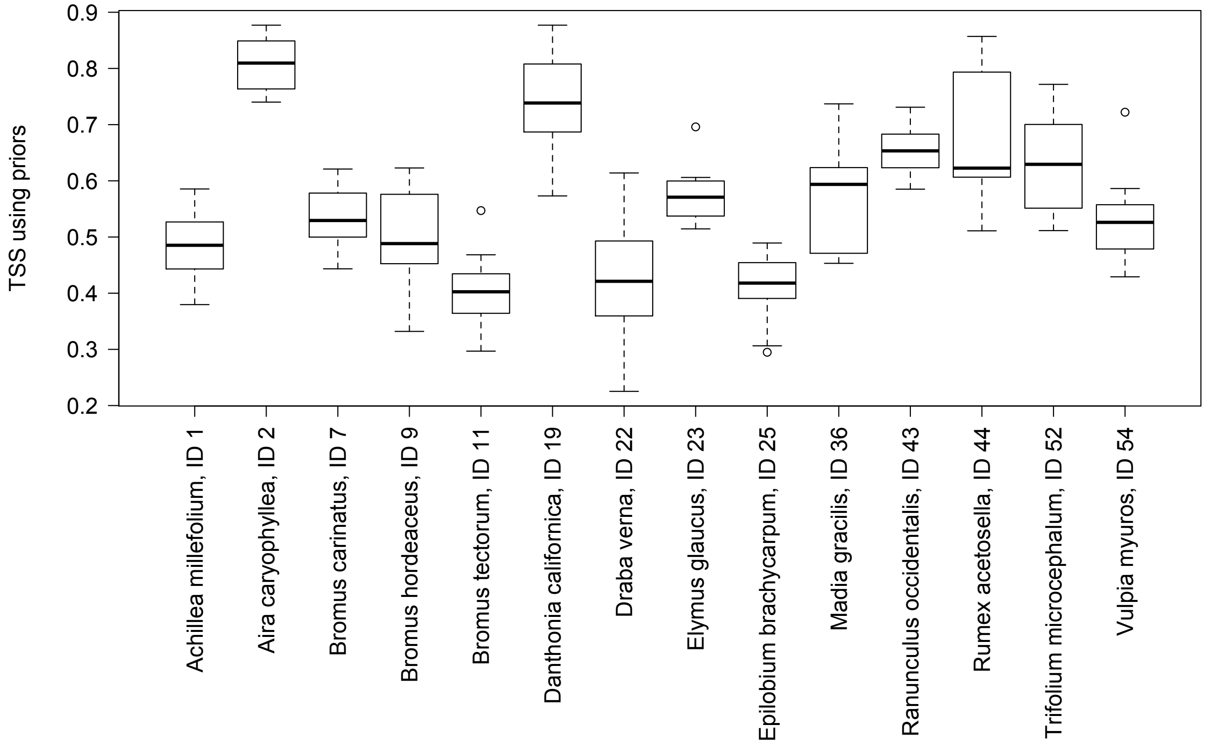

Figure S1: TSS scores for the 14 focal species without biotic interactions in SDMs.

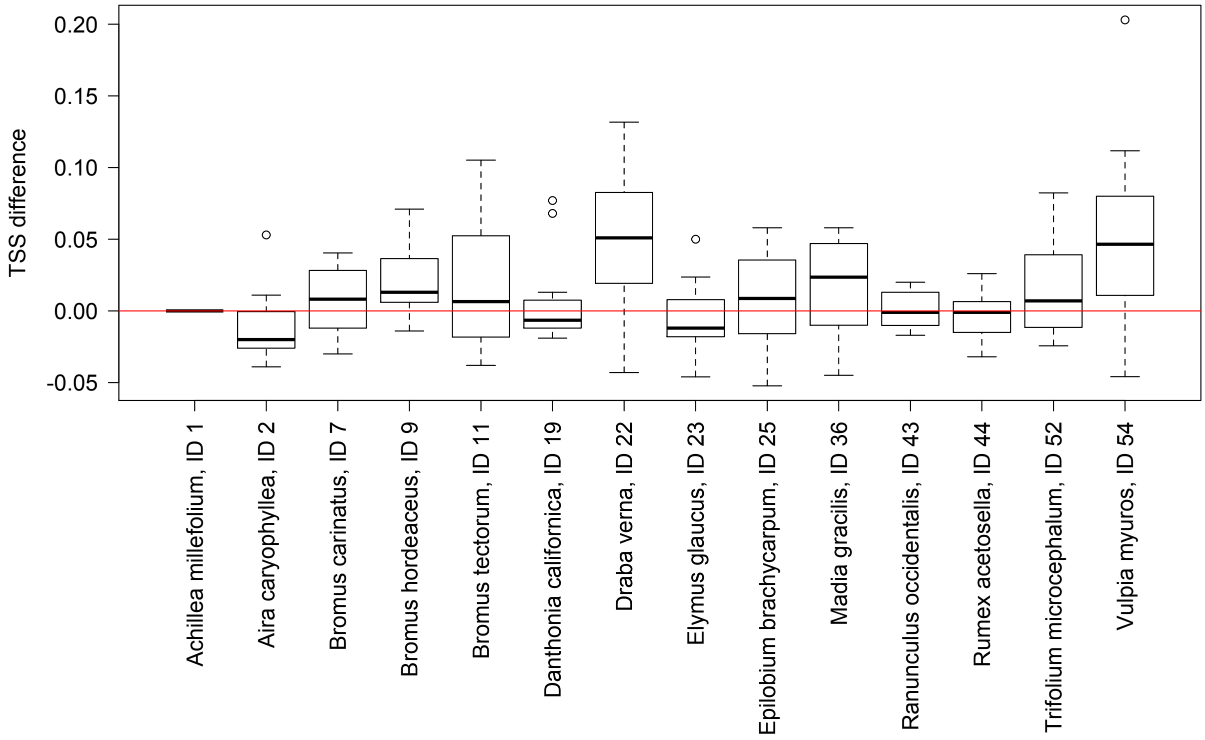

Figure S2: Increases and decreases in TSS scores for the 14 focal species when including biotic interactions in SDMs.

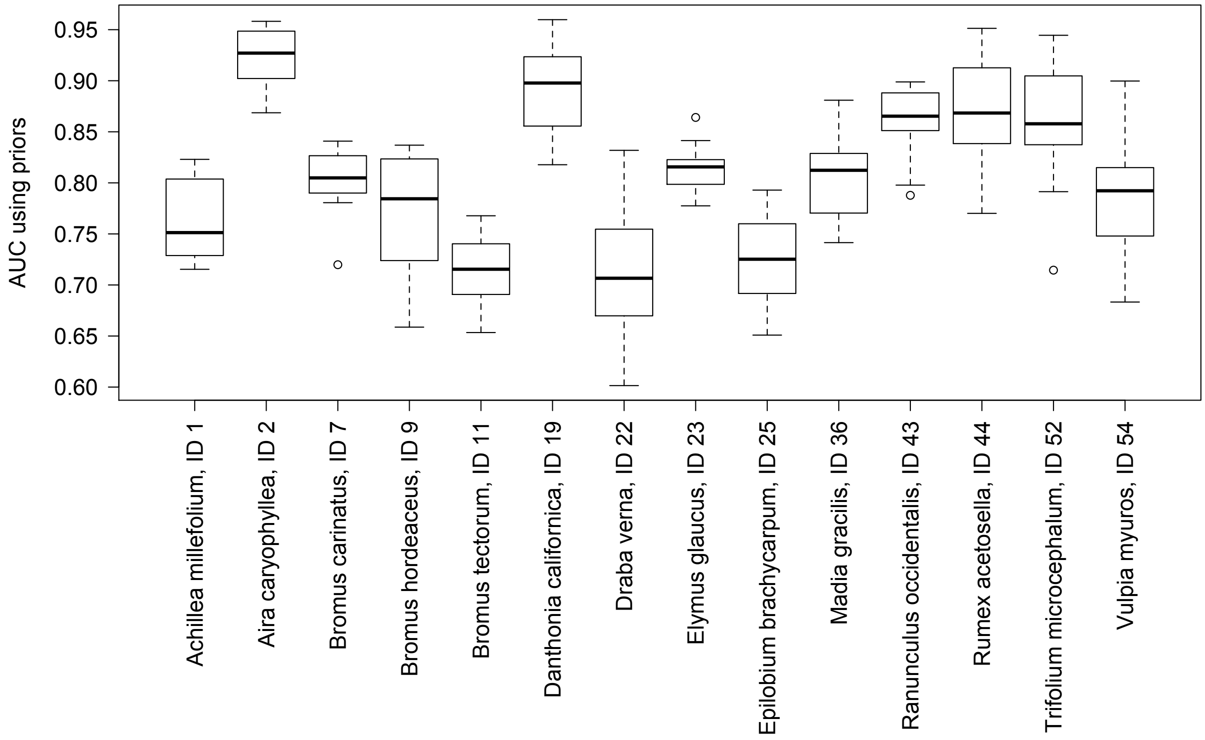

Figure S3: AUC scores for the 14 focal species without biotic interactions in SDMs.

Figures S4–S17. How to interpret colours in the following species range maps.

We considered species distribution model (SDM) outputs for the western USA at a resolution of $\sim$ 800m $\times$ 800m grid cells. The output of SDMs without biotic interactions and shared habitat suitability relationships is shown in bottom-left panels: habitat suitability values were transformed to a binary—green (■) = "present" and grey (■) = "absent"—species range using the maxSSS threshold and allowing for dispersal.

The effect of including biotic interactions and shared habitat suitability relationships in SDMs for 2010 ("present day") is highlighted in top-left panels: additional "present" locations are in yellow (■) and additional "absent" locations are in cyan (■).

The effect of environmental change between 2010 and 2050 on species ranges predicted using SDMs without biotic interactions and shared habitat suitability relationships is highlighted in bottom-right panels: lost geographical range is in magenta (■).

The effect of environmental change between 2010 and 2050 on species ranges predicted using SDMs with biotic interactions and shared habitat suitability relationships is highlighted in top-right panels: compared to SDMs for 2050 *without* biotic interactions and shared habitat suitability relationships, additional "present" locations are in yellow (■) and additional "absent" locations are in cyan (■); and lost geographical range compared to SDMs for 2010 *with* biotic interactions and shared habitat suitability relationships is in magenta (■), with "absent" locations that are predicted as being "present" using SDMs for 2050 *without* biotic interactions and shared habitat suitability relationships in blue (■).

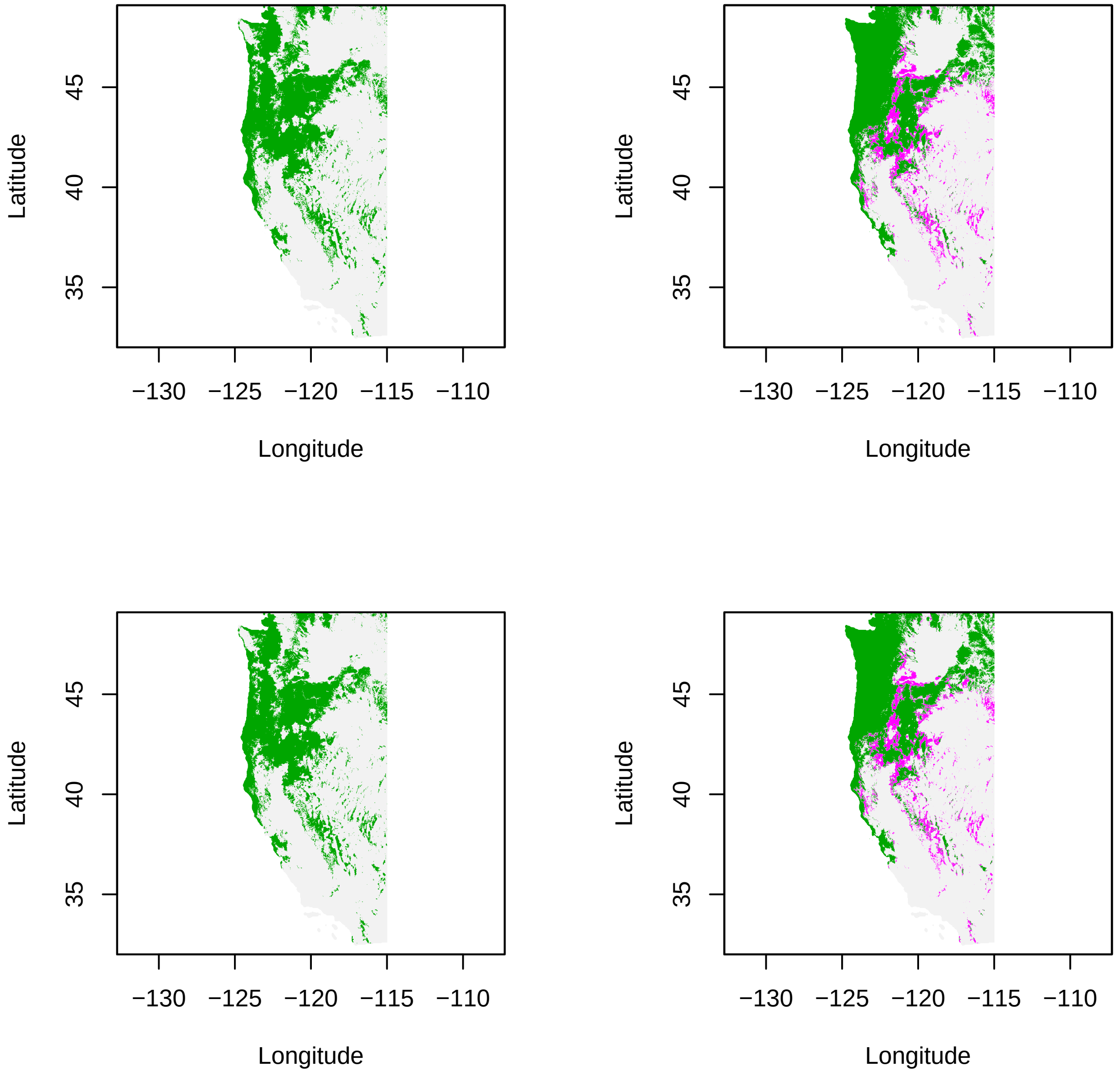

Figure S4: Predicted change in the geographical range of *Achillea millefolium* (perennial forb, species ID 1) between 2010 (left) and 2050 (right) using SDMs with (top) and without (bottom) biotic interactions and shared habitat suitability relationships. See Page 22 for a description of the colour scheme.

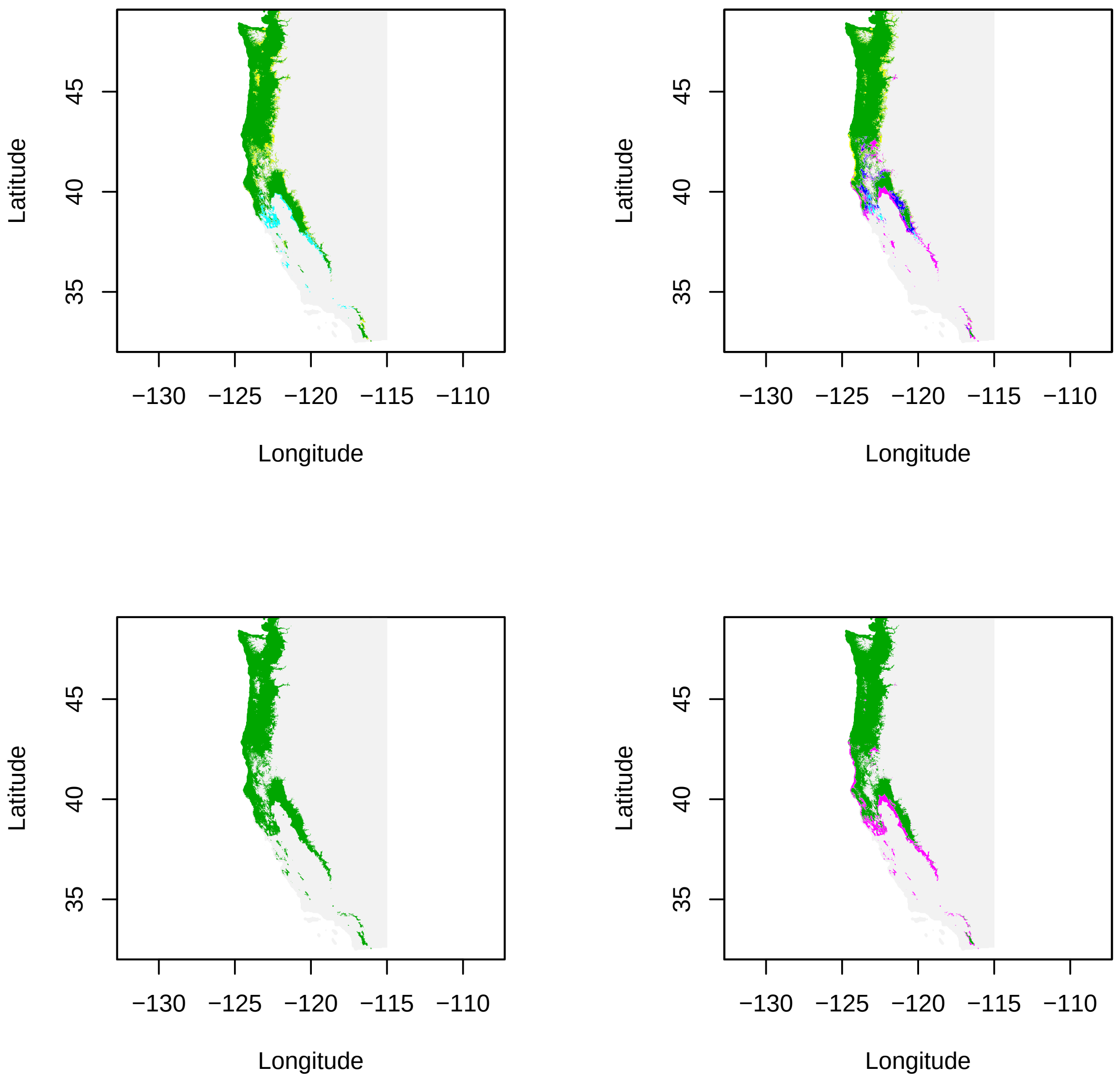

Figure S5: Predicted change in the geographical range of *Aira caryophyllea* (annual grass, species ID 2) between 2010 (left) and 2050 (right) using SDMs with (top) and without (bottom) biotic interactions and shared habitat suitability relationships. See Page 22 for a description of the colour scheme.

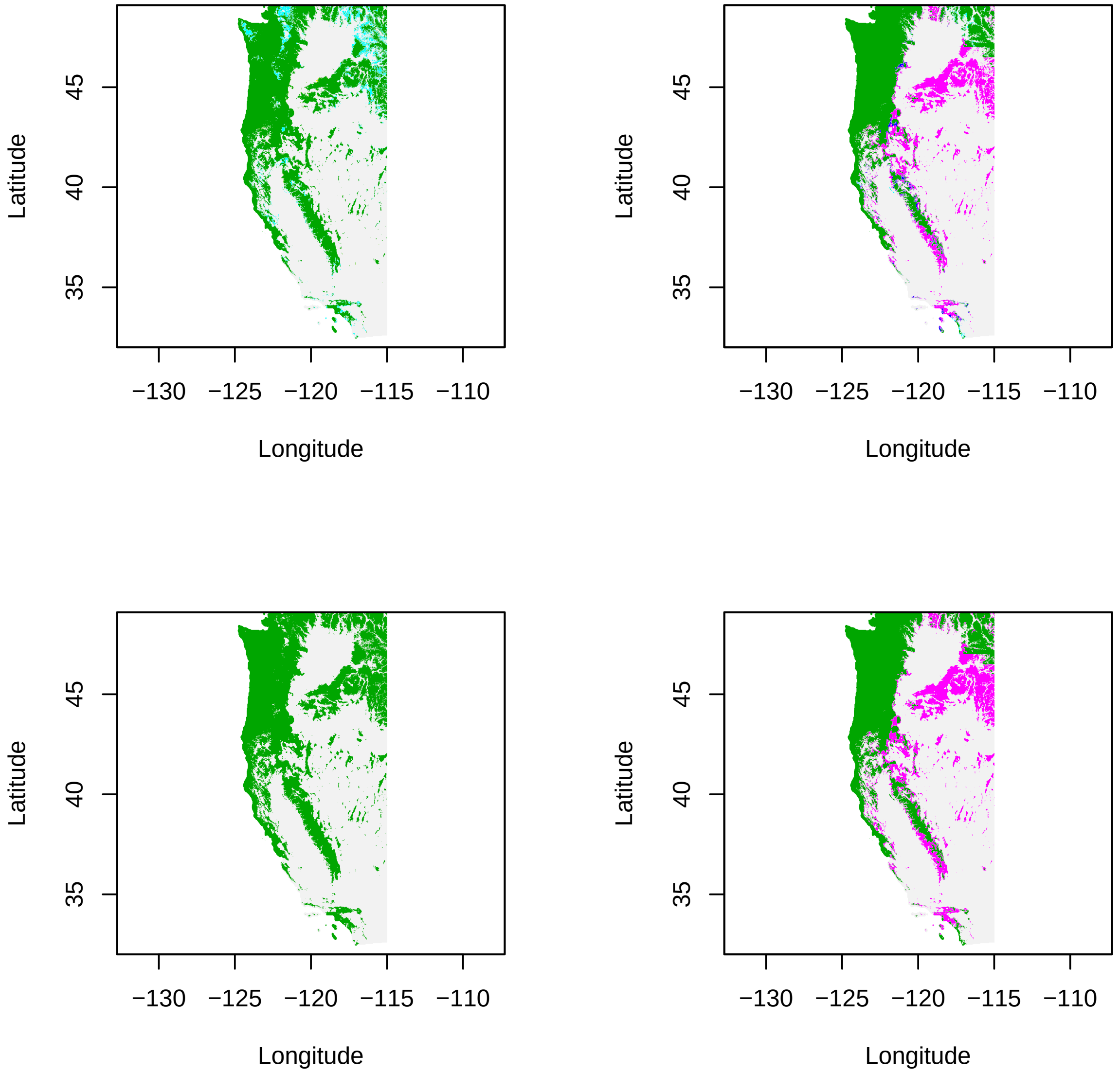

Figure S6: Predicted change in the geographical range of *Bromus carinatus* (annual grass, species ID 7) between 2010 (left) and 2050 (right) using SDMs with (top) and without (bottom) biotic interactions and shared habitat suitability relationships. See Page 22 for a description of the colour scheme.

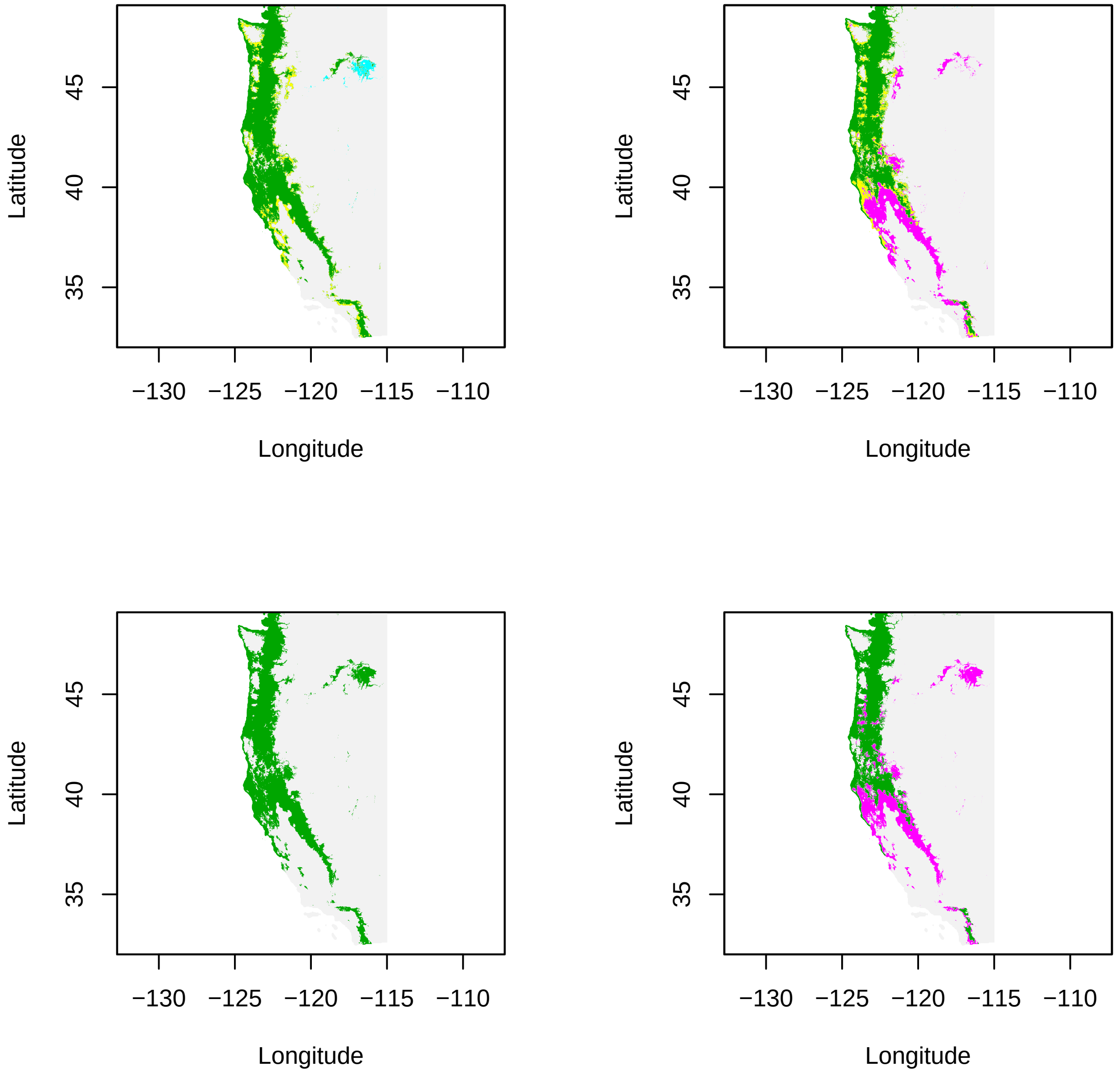

Figure S7: Predicted change in the geographical range of *Bromus hordeaceus* (annual grass, species ID 9) between 2010 (left) and 2050 (right) using SDMs with (top) and without (bottom) biotic interactions and shared habitat suitability relationships. See Page 22 for a description of the colour scheme.

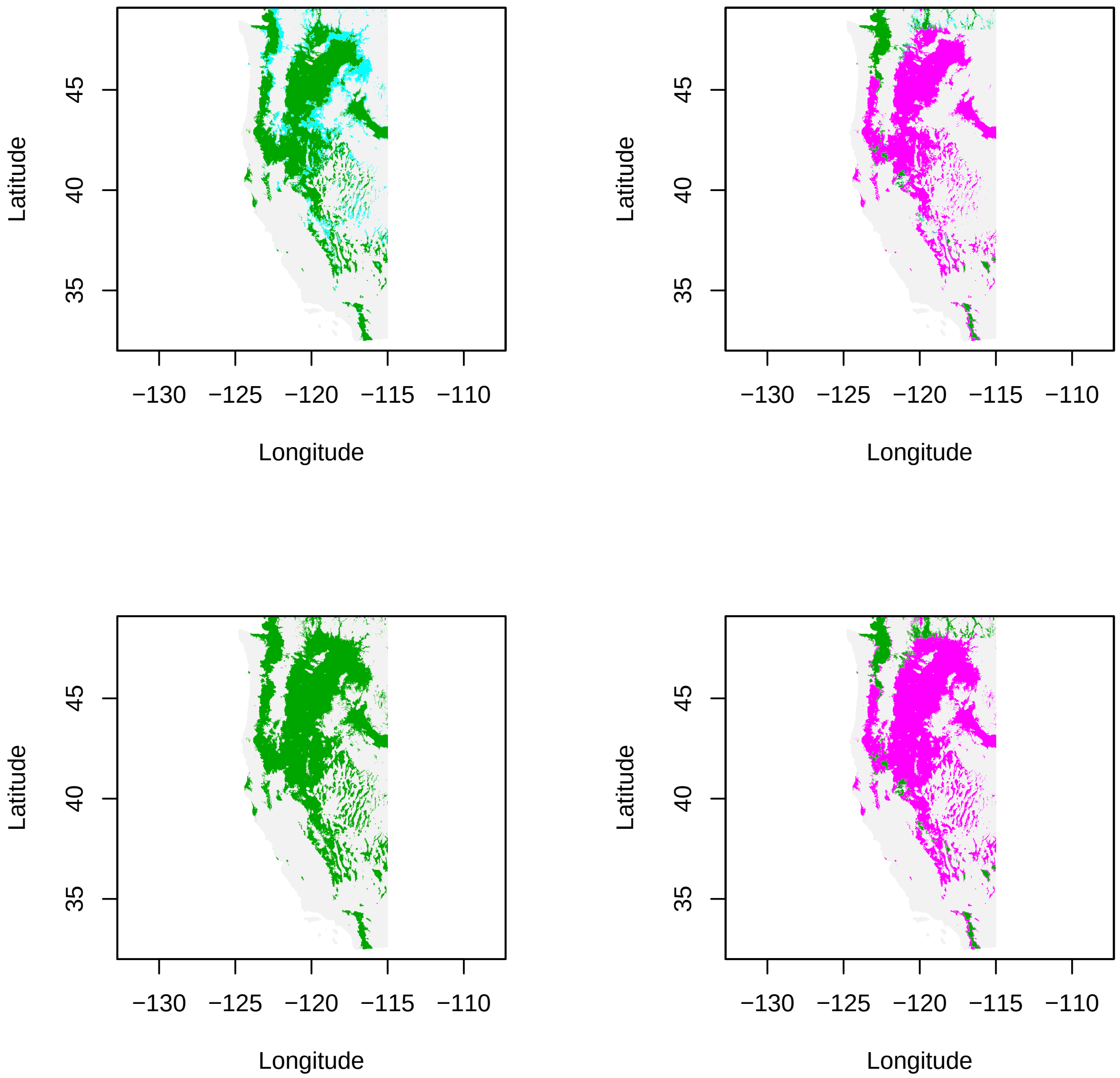

Figure S8: Predicted change in the geographical range of *Bromus tectorum* (annual grass, species ID 11) between 2010 (left) and 2050 (right) using SDMs with (top) and without (bottom) biotic interactions and shared habitat suitability relationships. See Page 22 for a description of the colour scheme.

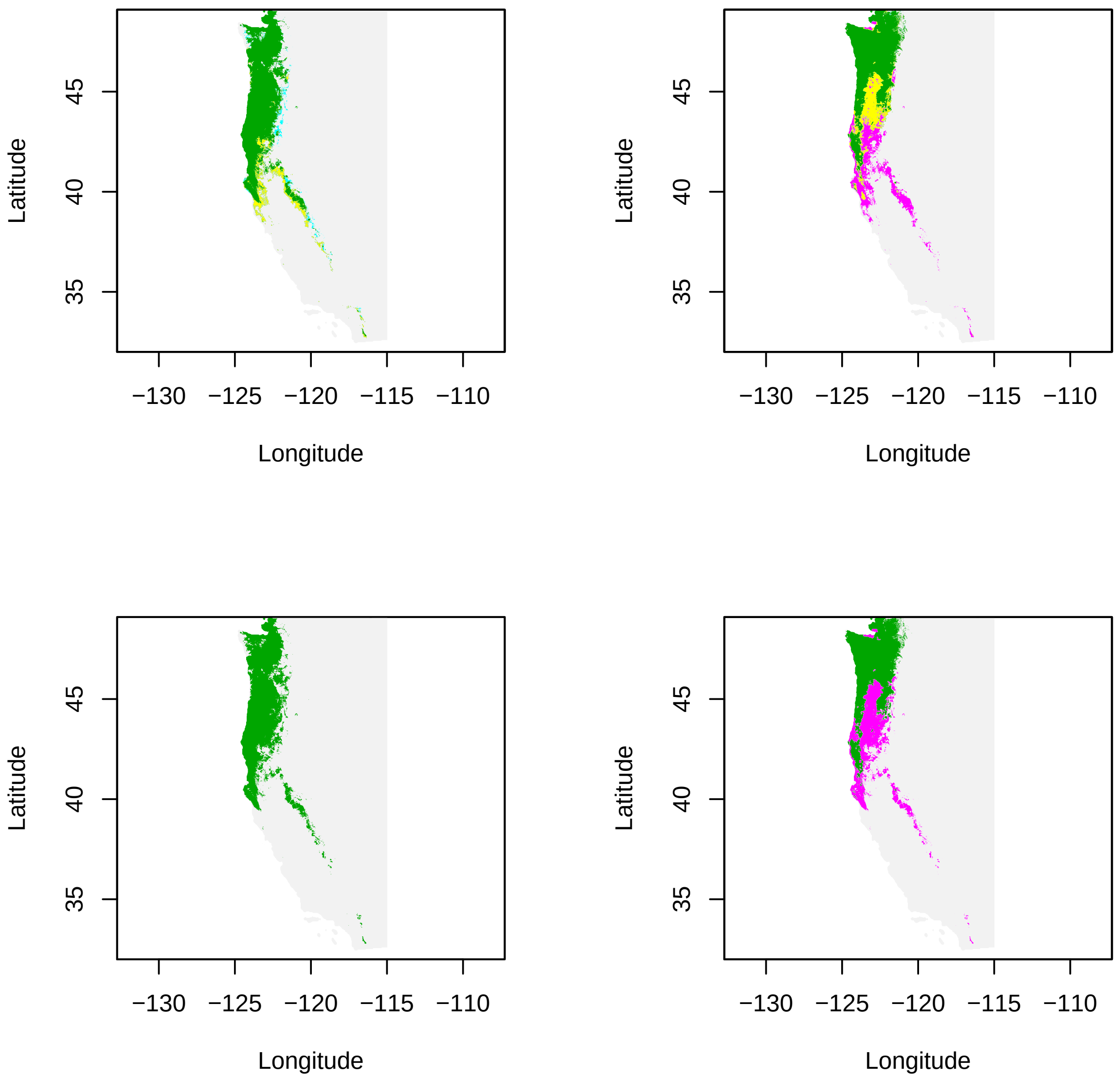

Figure S9: Predicted change in the geographical range of *Danthonia californica* (perennial grass, species ID 19) between 2010 (left) and 2050 (right) using SDMs with (top) and without (bottom) biotic interactions and shared habitat suitability relationships. See Page 22 for a description of the colour scheme.

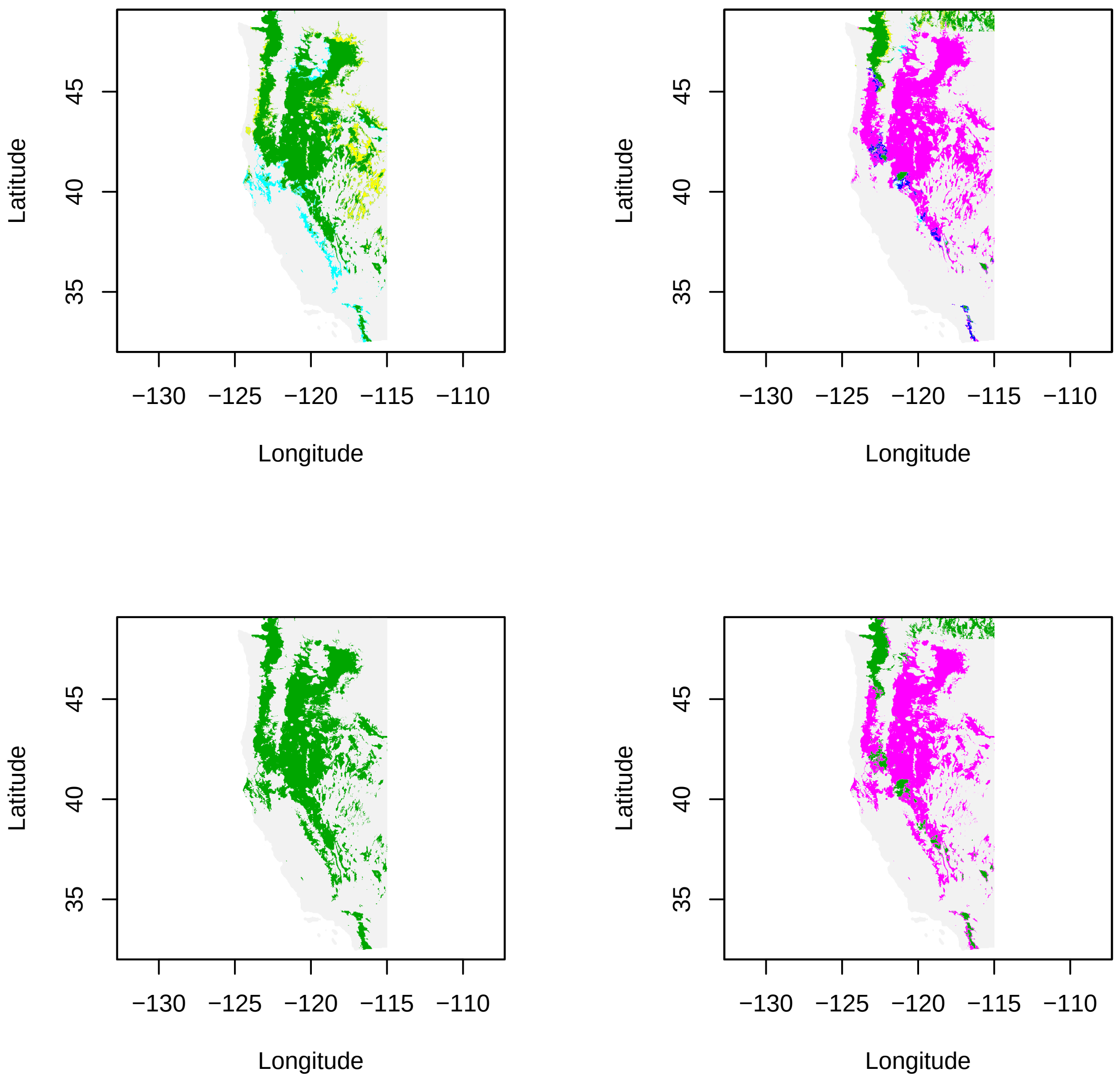

Figure S10: Predicted change in the geographical range of *Draba verna* (spring forb, species ID 22) between 2010 (left) and 2050 (right) using SDMs with (top) and without (bottom) biotic interactions and shared habitat suitability relationships. See Page 22 for a description of the colour scheme.

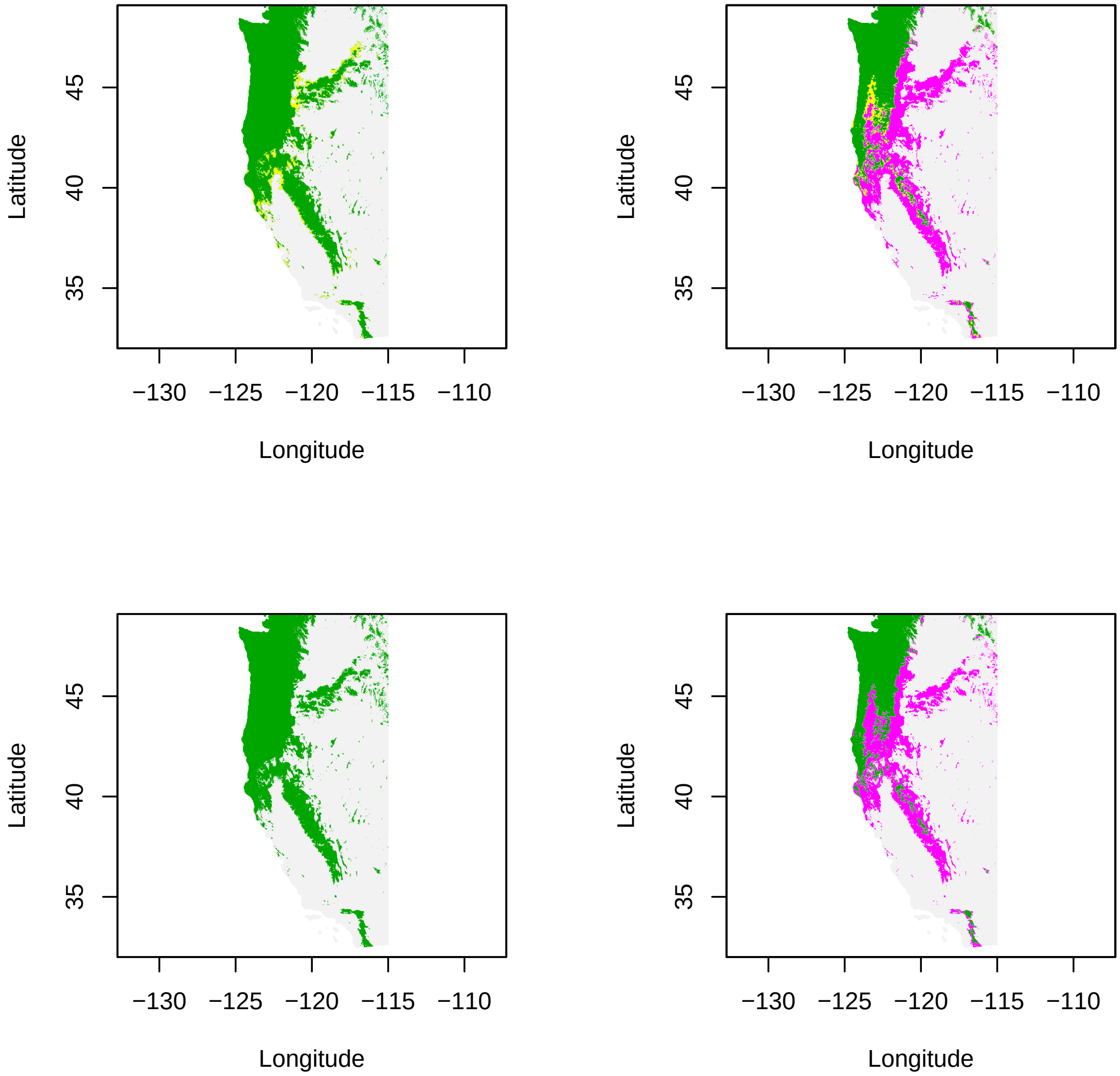

Figure S11: Predicted change in the geographical range of *Elymus glaucus* (perennial grass, species ID 23) between 2010 (left) and 2050 (right) using SDMs with (top) and without (bottom) biotic interactions and shared habitat suitability relationships. See Page 22 for a description of the colour scheme.

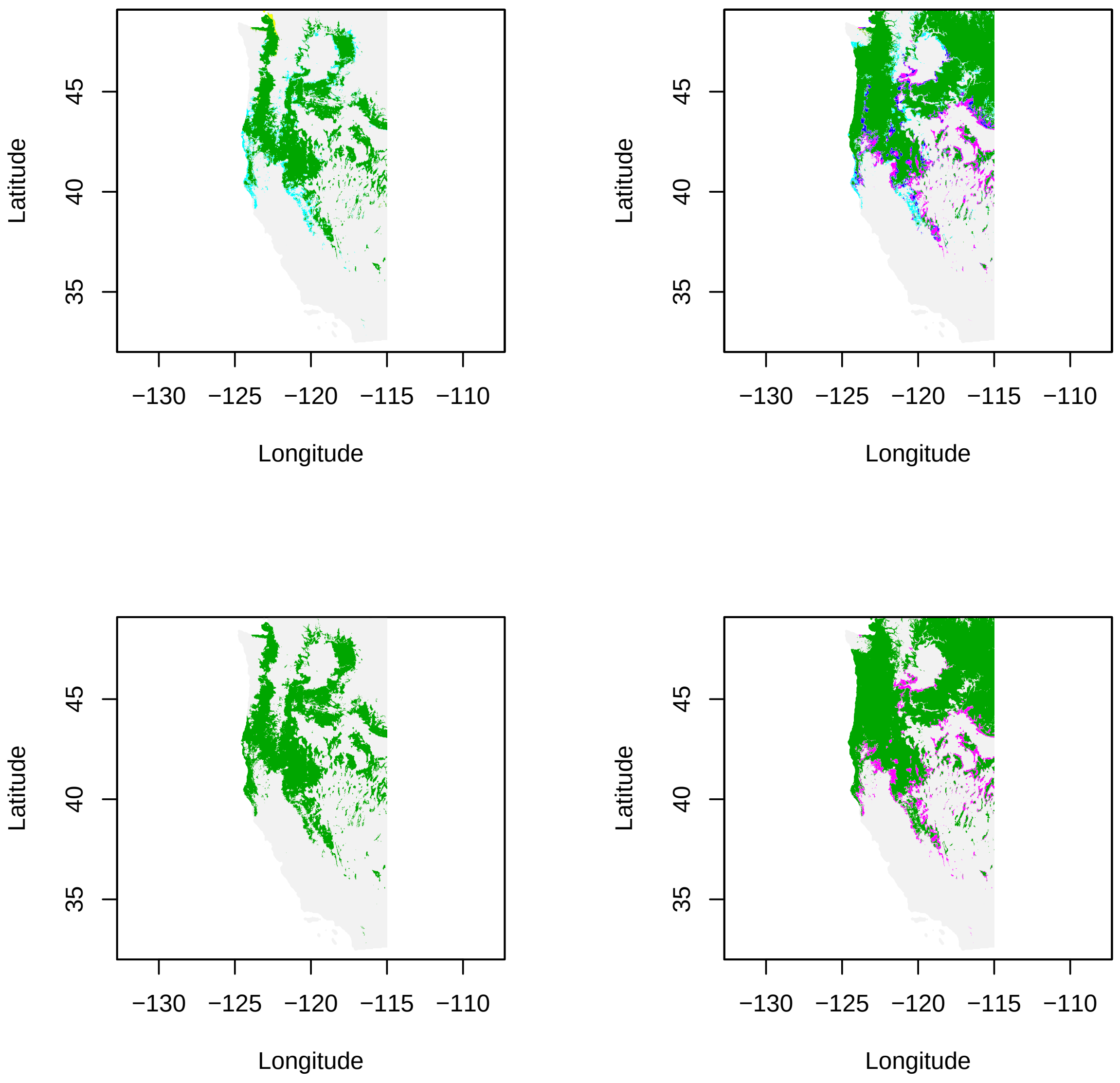

Figure S12: Predicted change in the geographical range of *Epilobium brachycarpum* (summer forb, species ID 25) between 2010 (left) and 2050 (right) using SDMs with (top) and without (bottom) biotic interactions and shared habitat suitability relationships. See Page 22 for a description of the colour scheme.

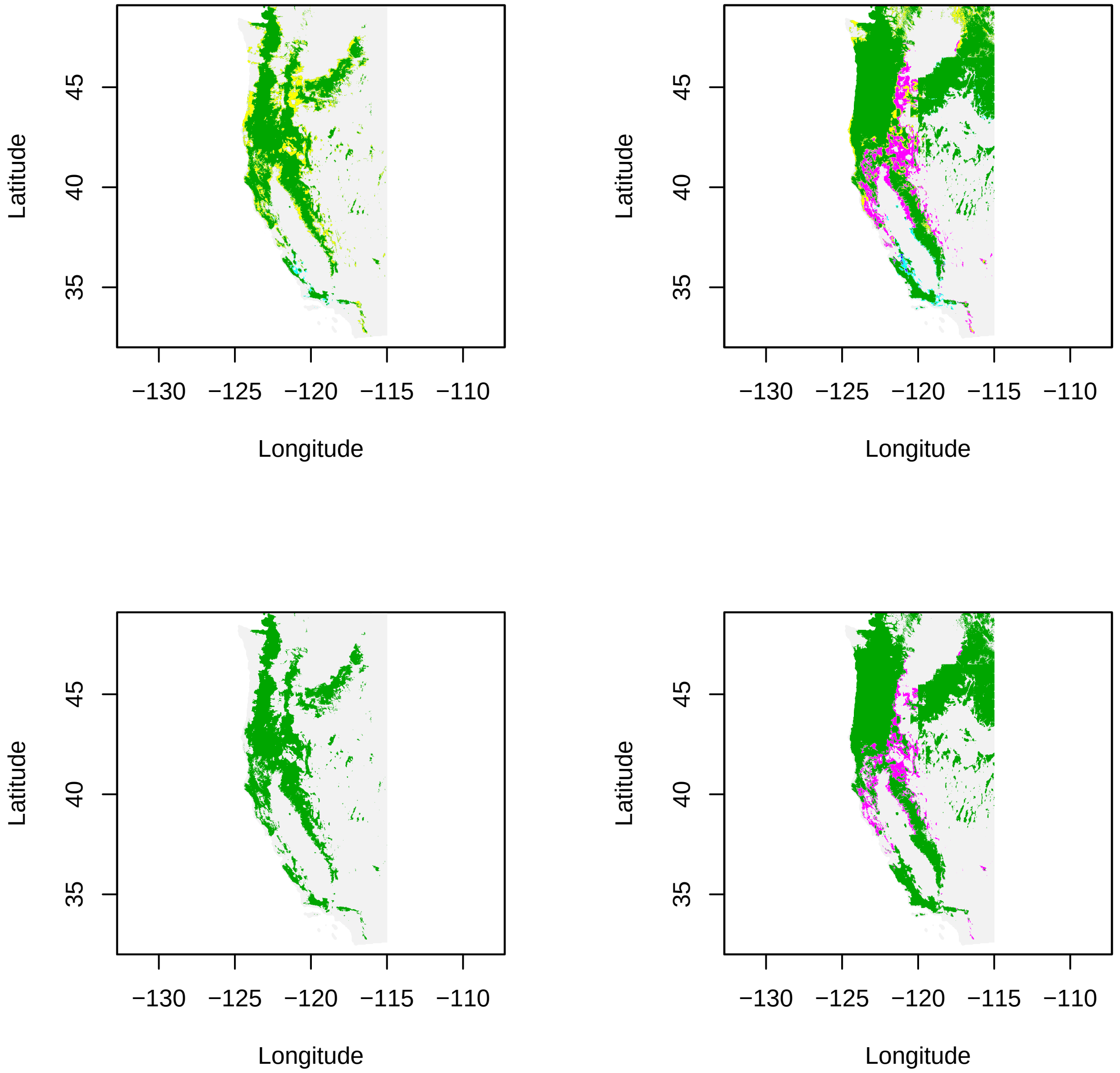

Figure S13: Predicted change in the geographical range of *Madia gracilis* (summer forb, species ID 36) between 2010 (left) and 2050 (right) using SDMs with (top) and without (bottom) biotic interactions and shared habitat suitability relationships. See Page 22 for a description of the colour scheme.

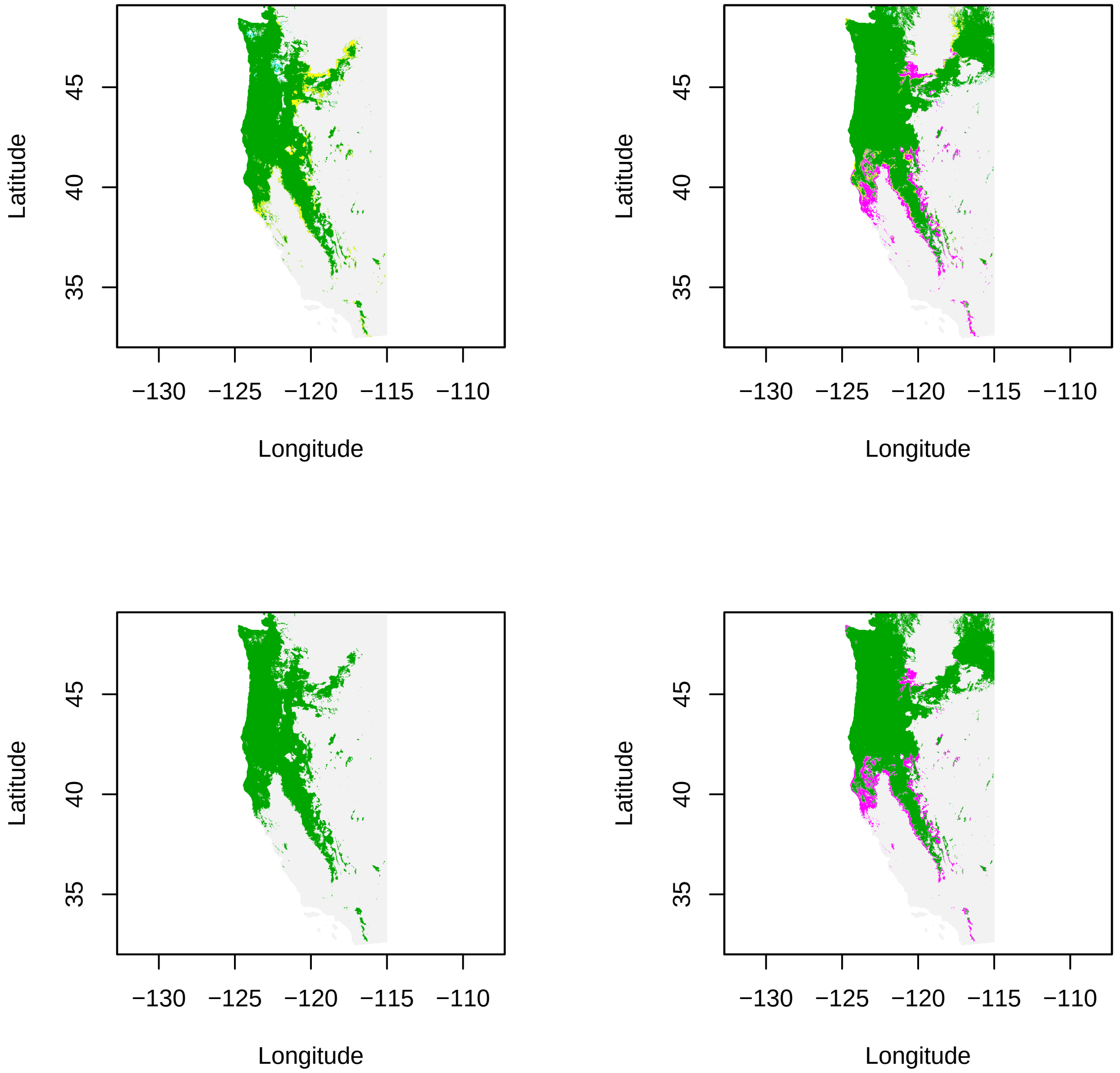

Figure S14: Predicted change in the geographical range of *Ranunculus occidentalis* (perennial forb, species ID 43) between 2010 (left) and 2050 (right) using SDMs with (top) and without (bottom) biotic interactions and shared habitat suitability relationships. See Page 22 for a description of the colour scheme.

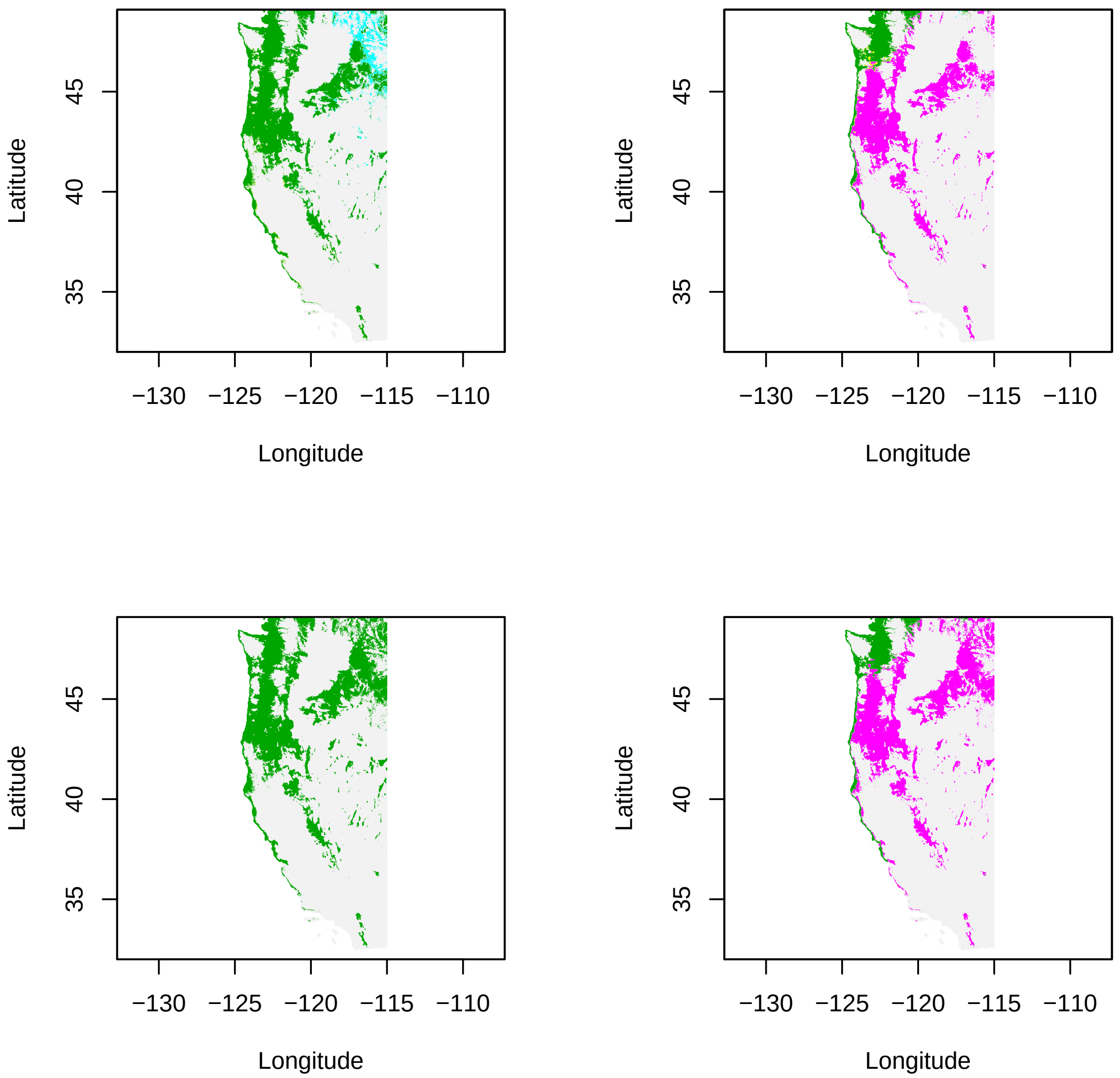

Figure S15: Predicted change in the geographical range of *Rumex acetosella* (perennial forb, species ID 44) between 2010 (left) and 2050 (right) using SDMs with (top) and without (bottom) biotic interactions and shared habitat suitability relationships. See Page 22 for a description of the colour scheme.

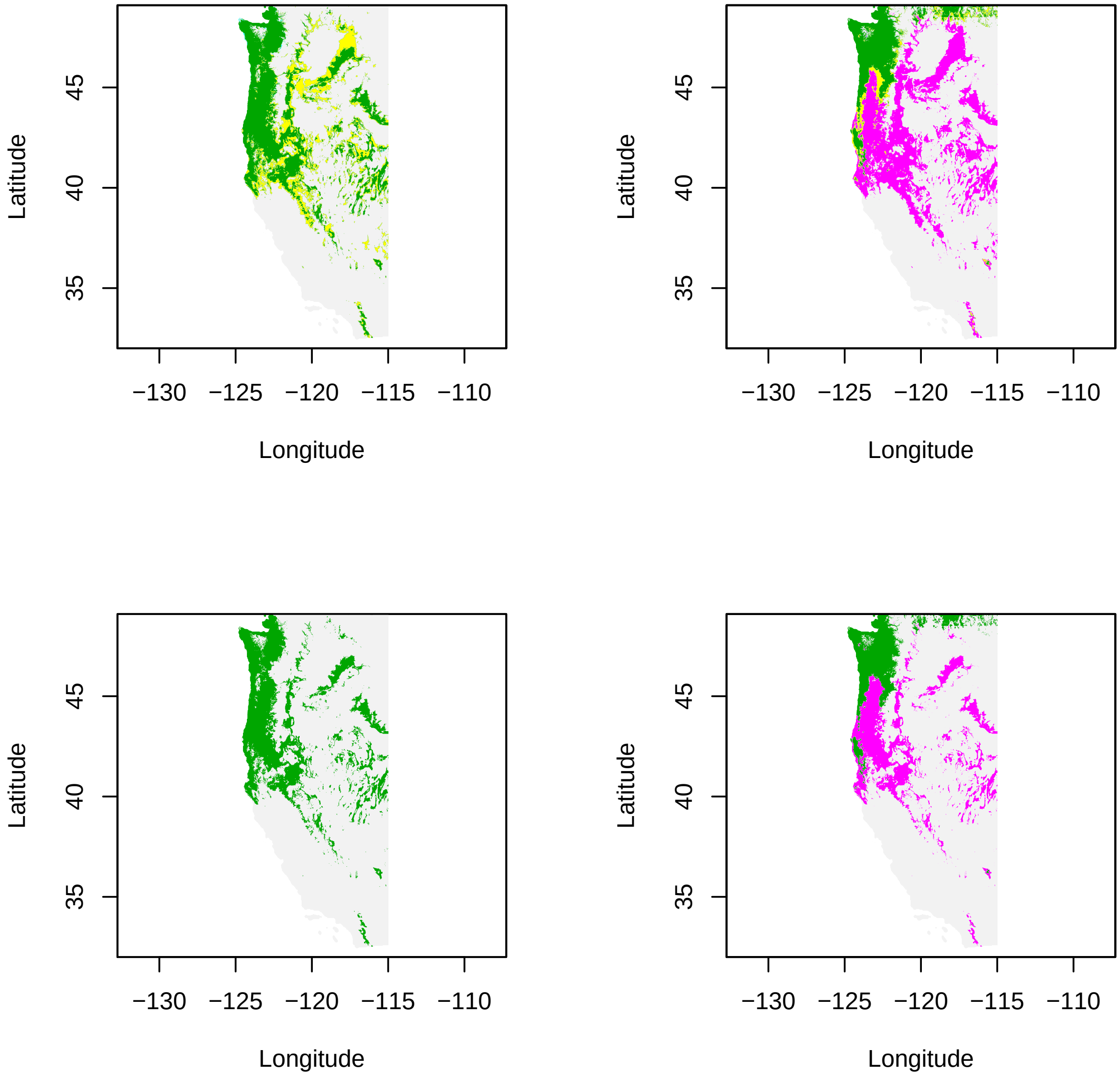

Figure S16: Predicted change in the geographical range of *Trifolium microcephalum* (nitrogen-fixing forb, species ID 52) between 2010 (left) and 2050 (right) using SDMs with (top) and without (bottom) biotic interactions and shared habitat suitability relationships. See Page 22 for a description of the colour scheme.

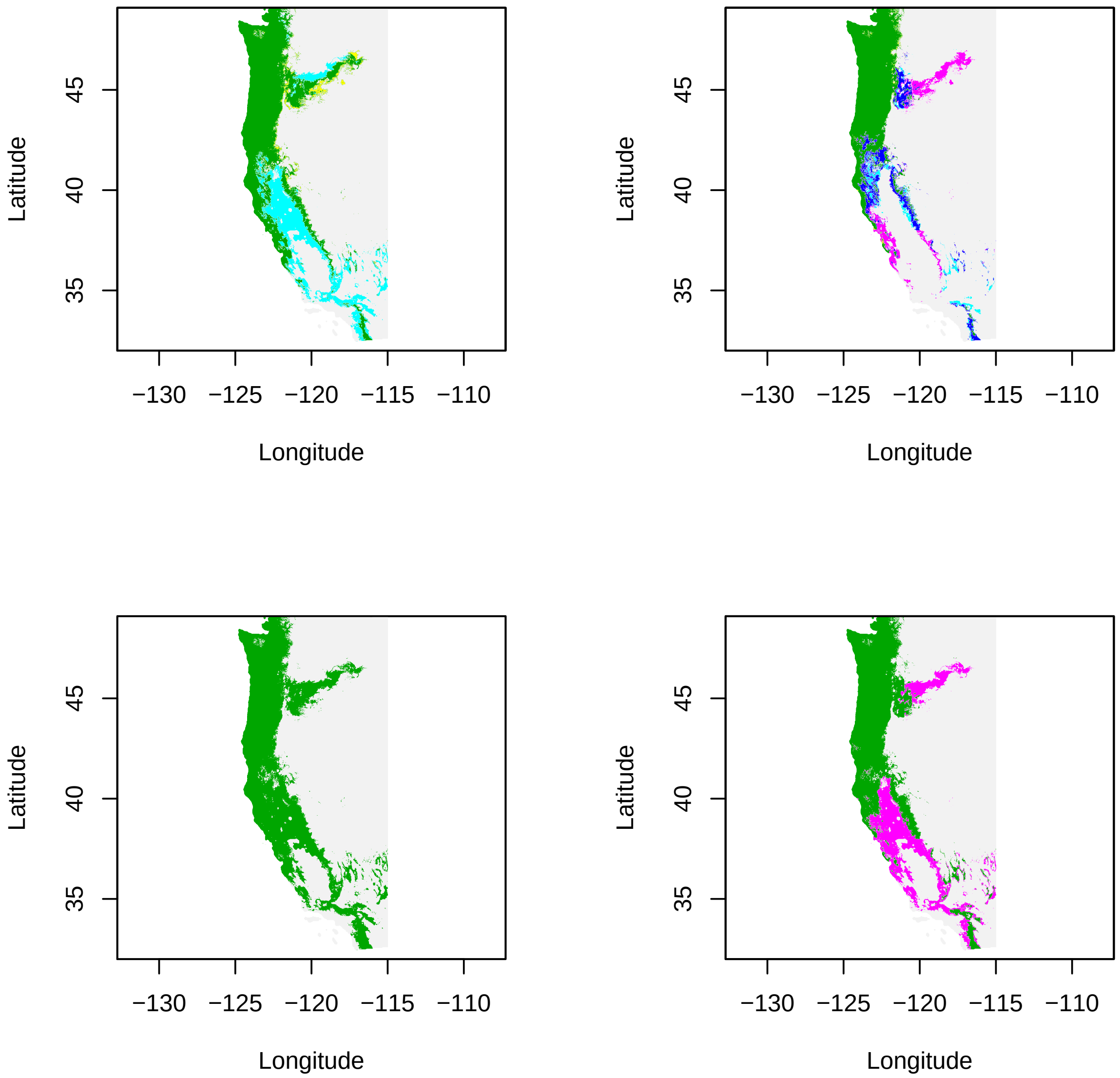

Figure S17: Predicted change in the geographical range of *Vulpia myuros* (annual grass, species ID 54) between 2010 (left) and 2050 (right) using SDMs with (top) and without (bottom) biotic interactions and shared habitat suitability relationships. See Page 22 for a description of the colour scheme.
